# Litter accumulation and fire risks show direct and indirect climate-dependence at continental scale

**DOI:** 10.1038/s41467-023-37166-9

**Published:** 2023-03-18

**Authors:** Mark A. Adams, Mathias Neumann

**Affiliations:** 1 School of Science, Computing and Engineering Technologies, Swinburne University of Technology, Hawthorn, Victoria Australia; 2 Institute of Silviculture, Department of Forest and Soil Sciences, University of Natural Resources and Life Sciences, Vienna, Austria

**Keywords:** Carbon cycle, Natural hazards, Ecosystem ecology

## Abstract

Litter decomposition / accumulation are rate limiting steps in soil formation, carbon sequestration, nutrient cycling and fire risk in temperate forests, highlighting the importance of robust predictive models at all geographic scales. Using a data set for the Australian continent, we show that among a range of models, most of the variance in litter mass over a 40-year time span can be accounted for by a parsimonious model with elapsed time, and indices of climate and litter quality, as independent drivers. Climate is an important driver of variation in both the species identity of dominant eucalypts and litter accumulation across the continent. Litter quality shows emergent properties of climate-dependence and contributes to explained variance. Nonetheless, elapsed time dominated explained variance in litter mass (up to 90%) at continental scale. Results provide guidance for future decomposition studies. Algorithms reported here can significantly improve accuracy and reliability of predictions of carbon and nutrient dynamics and fire risk.

## Abstract (web summary)

Compiled data on litterfall and litter in eucalypt forests and woodlands for the Australian continent shows that litter mass can be robustly predicted using just three independent variables – time, aridity and litterfall quality

## Introduction

A large proportion of terrestrial net primary production (NPP) is added to the litter layer through aboveground litterfall (e.g. >30% of annual NPP in European forests^1^, also^2^). Subsequently, litter either accumulates (until burnt; often too many times the mass of annual litterfall), decomposes to CO_2_, becomes stabilised organic matter added to the soils beneath, or is transferred in dissolved form^3^ (Supplementary Fig 1). Each of these fates has global significance owing to the size of the involved carbon fluxes. Current biogeochemical and Earth system models for carbon storage in the litter, for its return to the atmosphere, and for its addition to humus/soil in stabilised forms (e.g.^4–6^), rely on understandings of decomposition almost entirely derived from foliage of single species. When decomposition of mixtures of leaves of different species are studied, commonly seen are large non-additive effects such as the transfer of nutrients which are not predictable from the analysis of decomposition of the individual species^7^. Foliage decomposition is typically studied using litter bags and such studies dominate the decomposition literature^8^. Based on these studies, controls on decomposition are mostly viewed as a ‘hierarchy’. At a global scale, climate (moisture, temperature) regulates decomposition. At regional scales, biota (and biotic interactions with climate), can become dominant proximal drivers^9–11^. The strongest biotic regulator of variation in decomposition is typically litter quality which is mostly assessed via species identity of foliage^5,12–15^. The temporal scale of litterbag decomposition studies mostly extend to periods of 1–3 years^16^, while a handful of studies at scales up to the continental have extended for up to a decade (e.g. LIDET^17^, CIDET^18^). Important findings are that (a) longer-term rates of decomposition are over-estimated by short-term studies^17–19^, (b) litter decomposition is seldom complete and (c) including or excluding important decomposer fauna via the size of litterbag mesh significantly affects measured rates of decomposition^20,21^.

Blair et al.^22^ reported strong interactions of soil fauna with the release of carbon and nitrogen in a major study of decomposition of foliage mixtures. More recently, Mori et al.^23^ revealed that differences in rates of decomposition of leaf mixtures to those of leaves of individual species could be as large as differences due to climate change. The decomposition of complex mixtures (foliage, bark, wood) remains poorly studied and is both a major gap in knowledge and a weakness in global models.

Importantly, accumulated litter (< 6mm diam.; ‘fine fuel’) is also a driver of wildfires owing to its mass (=fine fuel, subject to particle size), its ease of ignition and combustion under dry conditions, and the strong relationship of fire intensity to consumed fuel^24^. While fine fuels are of somewhat reduced significance to fire behaviour under extreme weather conditions (e.g. low humidity, strong winds, high temperatures), accumulated litter remains a primary target for management interventions designed to help fire suppression^25^.

Recognition of the limits of litterbag studies in reflecting the decomposition of complex litter layers in the field has led to strong calls for the establishment of long-term studies of litter accumulation^26^. Such long-term studies are also critical to quantifying fire risks. Measuring the mass of litter remaining in a forest after a given period, is fully analogous to measuring the mass of litter remaining within litter bags, with the addition of annual litter inputs.

Here we use a continental-scale data set on litter accumulation to address significant gaps in global knowledge of litter decomposition and their consequences for currently used models of carbon biogeochemistry (e.g.^27,28^) and fire risk (e.g.^29,30^). The collated data and this study are facilitated by fire regimes which ensure the litter layers of many, if not most, eucalypt forests and woodlands burn at moderate-high frequency (e.g. 40–100 years). We use the data to develop de novo models and test prevailing theory, derived from studies using litterbags, as to regulation of litter decomposition and the assumptions implicit in global models. Current theory predicts that the decomposition and accumulation of mixtures of litter (leaves, bark, twigs and fruiting bodies) should be regulated by climate and litter quality. This is our primary hypothesis. More formally, the outcome of litterfall inputs and decomposition – the mass of litter - should be related to the elapsed period of accumulation and the known influences of moisture, temperature and quality. In practice, effects of moisture and temperature have been modelled both independently and combined in indices of climate. Climate indices have a long history of use in studies of decomposition and turnover (e.g.^31^) including in eucalypt forests^32^. We used a metric for quality (Methods) based on relative inputs of different types of litter (with different quality) to the overall litter mixture. We examined the climate-dependence of quality as part of our overall analysis, with a focus on variation as a function of scale (scale-dependent or scale-independent; *sensu* Bradford et al.^10^).

## Results

### Characteristics of litter and litterfall

**Fig. 1:**
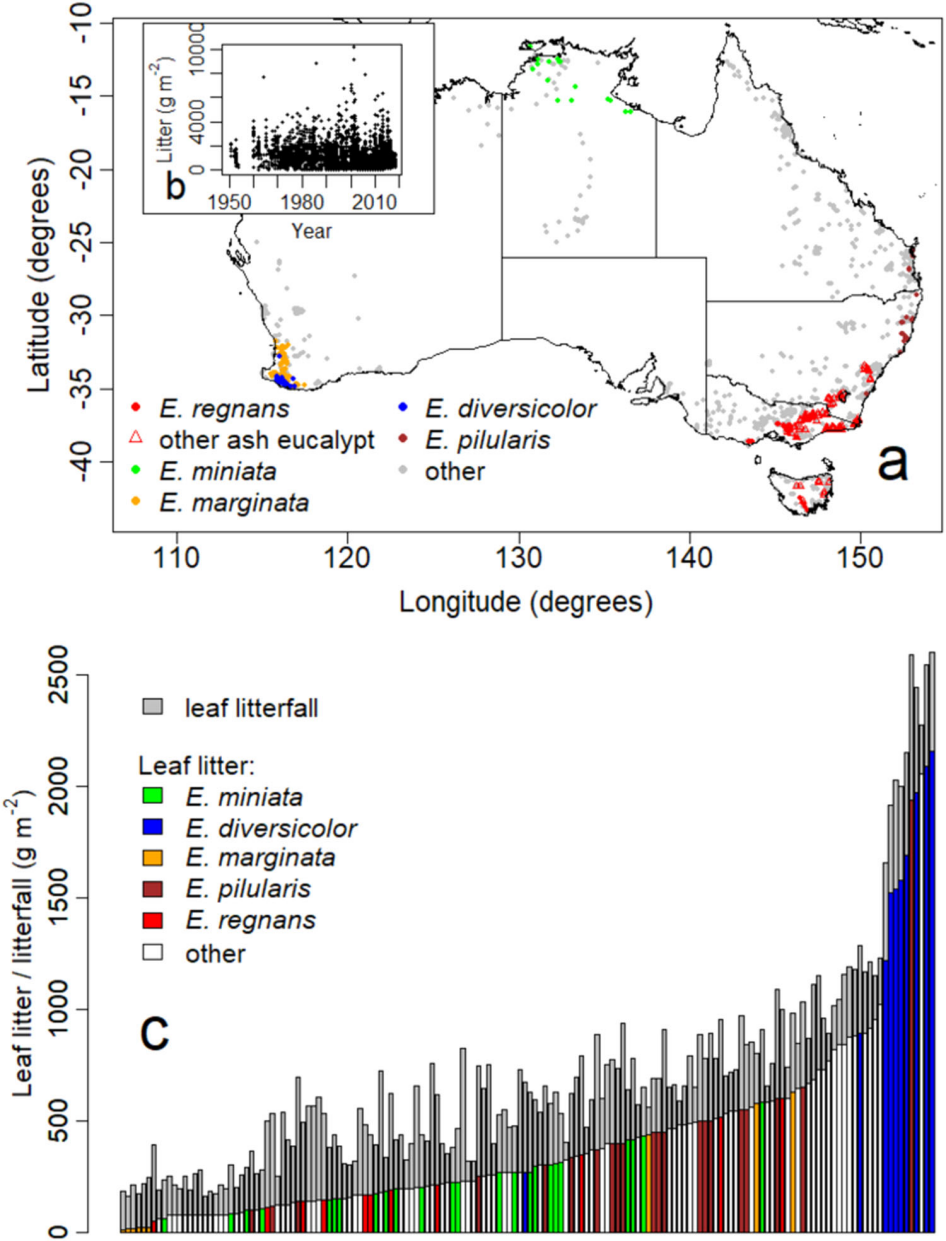
Leaf litter and litterfall observations for individual locations on the Australian continent. **a** Geographic locations of observations. **b** Relationship of observations for litter to year of sampling. **c** Mass of leaf litter or leaf litterfall for forests and woodlands in rank order of leaf litter mass. Base map after Neumann et al.^69^ prepared using R software^73^.

Geographic distribution of data for litter and litterfall broadly reflected continental distributions of forests and woodlands dominated by eucalypts (Fig. 1a, Table 1). Sampling year (as opposed to time elapsed) had no effect on recorded litter mass (Fig. 1b). Litterfall in all eucalypt forests and woodlands varied considerably from year to year. For example, annual leaf litterfall (the largest component) for any given forest in any given year is unrelated to long-term leaf litter accumulation (Fig. 1c). Forest types with small accumulations of litter (i.e. <250 g m^−2^) can have similar litterfall to those where litter mass ranges between 1000 and 2000 g m^−2^ (Fig. 1c).

**Table 1:** Summary of climate, location and sample size for litter data from all Australian forests and woodlands, for all eucalypt-dominated forest and woodlands, and for five representative communities

Biotic community	*n*	Annual precipitation (mm)		Mean daily temperature (^o^C)		Latitude (^o^S)	Longitude (^o^E)
		Range	Mean	Range	Mean	Range	Range
All data^a^	3914	204–4254	1130	5.6–28.5	16.9	10.9–43.4	114.3–153.5
All eucalypts^b^	2673	242–3909	1070	5.6–28.5	15.7	11.7–43.4	115.1–153.5
*E. regnans*	98	1087–1908	1470	9.1–13.2	10.7	37.4–43.3	143.5–146.9
*E. pilularis*	108	1152–1872	1447	15.6–21.1	18.3	25.4–35.4	150.4–153.3
*E. diversicolor*	160	656–1242	1119	14.7–16.3	15.3	32.8–34.8	115.8–116.9
*E. marginata*	547	580–1191	1028	14.5–18.8	15.7	31.8–34.8	115.5–117.5
*E. miniata*	58	837–1666	1501	26.3–27.5	27.2	11.7–16.1	130.7–136.6

^a^Includes non-eucalypt dominated forests and woodlands such as wet tropical forests in coastal northern Queensland, semi-arid *Casuarina* and *Callitris* dominated woodlands of inland Australia, and areas of coniferous forest (e.g. Tasmania, Queensland).

^b^Includes forests and woodlands dominated by species from the genera *Eucalyptus, Angophora, Corymbia*.

Our analysis focused on five well-described eucalypt communities (forests and woodlands representing ~40% of all litter observations) that collectively span the continent and for which there is a good spread of observations across 40 years since previous fire (Table 1). We also aggregated data for two vegetation formations of large geographic extent (Grassy forests, Grassy woodlands; Table 1: Supplementary Table 1: Supplementary Fig 2), and separately for ‘Ash forests’ based on genetic similarity (data available for Ash forests = *E. regnans*, *E. delegatensis*, *E. sieberi*) - a further 30% of all litter data. Data aggregated in this fashion can have large ranges (and outliers) due to variations in productivity of eucalypt communities within such groupings (e.g. Shrubby forests in Supplementary Fig 2).

### Continental scale patterns of litter accumulation

**Fig. 2:**
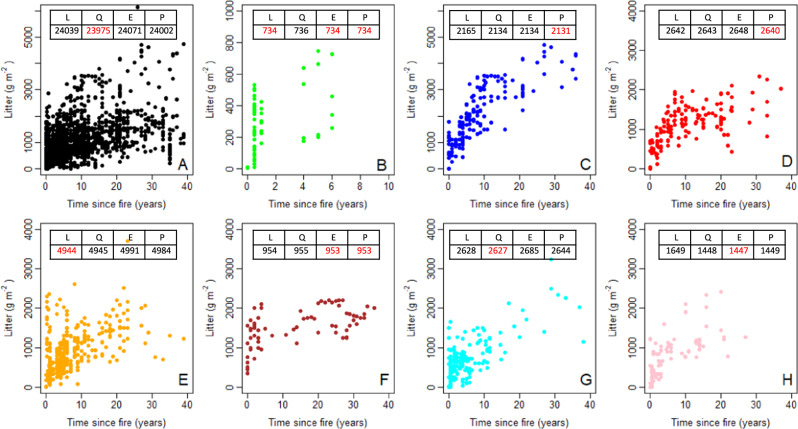
Relationship of litter mass to T_sf_ (time since fire) for eucalypt forests and woodlands. Panel **A** = All *Eucalyptus*. Panel **B** = *E. miniata*, **C** = *E. diversicolor*, **D** = Ash forests, E = *E. marginata*, **F** = *E. pilularis*, **G** = Grassy forests, **H** = Grassy woodlands. For each data subset, we show Akaike’s Information Criterion (AIC) for each of the fitted functions (L = Linear, Q = Quadratic, E = Exponential, P = Power). Best-fit models are highlighted in red. Further details of aggregated data are provided in Supplementary Fig 2 and Methods.

Data on litter accumulation in all eucalypt forests and woodlands across the Australian continent suggests continual increase with time (up to our imposed 40-year limit; Fig. 2A). Similarly, selected individual forest or woodland communities (Figs. 2B, 2C, 2E, 2F), and forests and woodlands aggregated via structure or genetics (Fig. 2D, 2G, 2H) show continual accumulation. With the exception of *E. miniata* communities (Fig. 2B), the data suggest that accumulation slows in all individual or aggregated communities (i.e. Fig. 2C–H). As examples, accumulation slows quickly in *E. pilularis* (Fig. 2F) but only slowly in *E. diversicolor* (Fig. 2C).

As expected, the availability of data declines as periods of elapsed time (time since fire) increase. Especially for *E. miniata, E. marginata* and *E. pilularis* communities, large spreads in litter mass in the first few years after fire are attributable to incomplete combustion of litter - in effect a contamination of post-fire with pre-fire litter. Based on AIC, power and polynomial (quadratic) functions generally better describe the data than linear or exponential functions (Fig. 2A–H). Data limitations (e.g. Fig. 2B) and issues of incomplete combustion (e.g. Fig. 2E, F) play a role in that assessment.

### Modelling litter accumulation

**Fig. 3:**
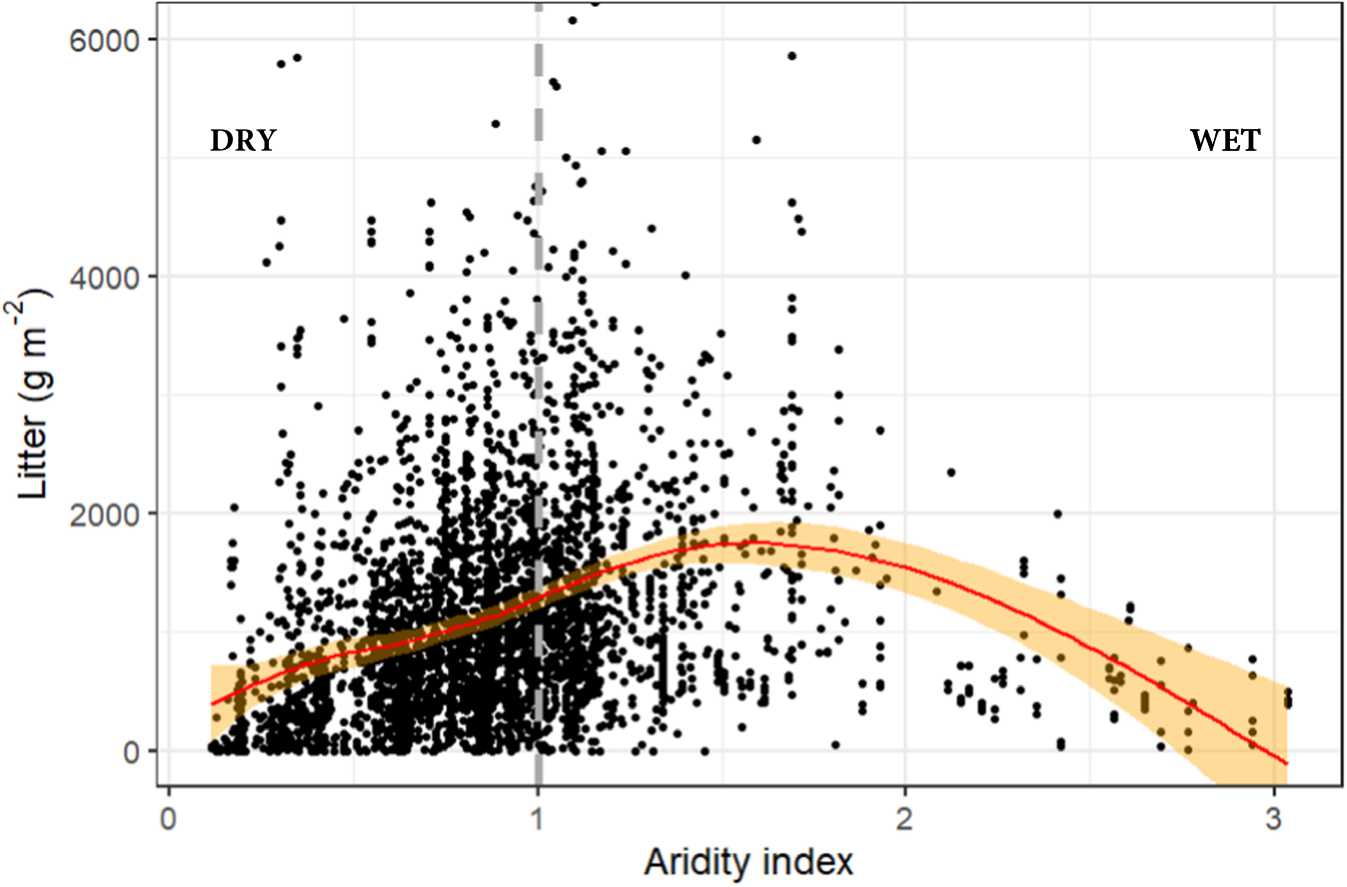
Relationship of litter mass (X_Tsf_) to Aridity Index (AI) for Australian forests and woodlands. AI = 1 (vertical dashed gray line) when precipitation and evapotranspiration are equal. Red line is moving average and orange shading represents the 0.999 confidence interval.

Following the extensive global literature, we also assessed climate and litter quality separately as major influences on litter accumulation. Climate has clear direct effects on litter accumulation across the Australian continent (Fig. 3). For any given sampling point and period of elapsed time (since previous fire; X_Tsf_), litter mass is non-linearly related to aridity index (AI). Measured X_Tsf_ reaches a maximum at ~AI = 1.5 (i.e. where precipitation exceeds evapotranspiration by 50%).

**Fig. 4:**
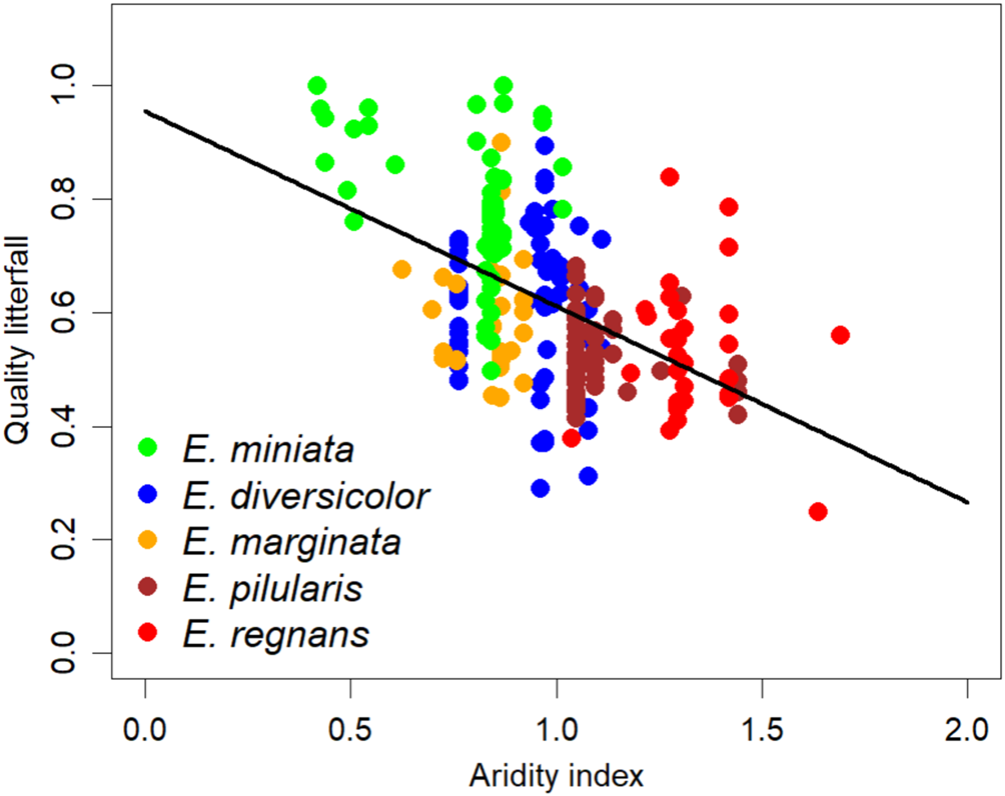
Relationship of litterfall quality (Q_lf_) to Aridity Index (AI) for five representative eucalypt communities. Q_lf_ = 1.009 − 0.387 AI. *P* < 0.001, *R*^*2*^ = 0.32.

**Fig. 5:**
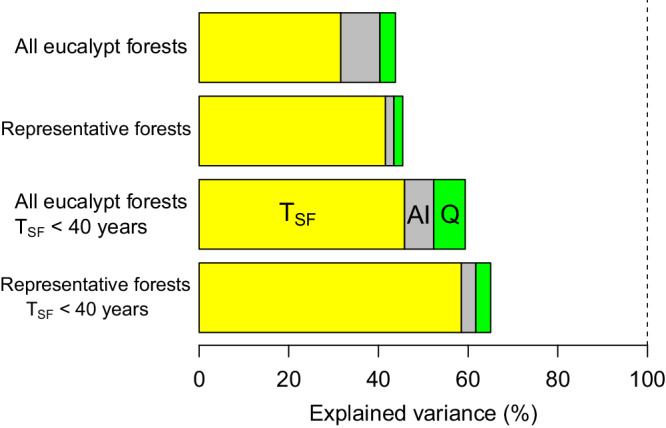
Contributions of independent variables to explained variance in best fit models. (Table 2).

The quality of litter for decomposition at any given sampling point was quantified as the proportion of leaves in annual litter inputs (i.e. leaf litterfall/total litterfall; Q_lf_). Leaves are of far better quality than wood or bark components (Supplementary Figs 3 and 4). Q_lf_ varies five-fold across the continent from ~0.2 to 1.0 and shows clear scale-dependence in relation to climate (Fig. 4). There is large variation within given forest types and the climate dependence of Q_lf_ is an emergent property. Summary statistics for AI and Q_lf_ for each of five selected communities are shown in Supplementary Fig 4. We thus included the direct (AI) and indirect (Q_lf_) climatic influences on litter accumulation with those of time, in the following more detailed analysis (Table 2, Fig. 5, Supplementary Tables 1–3).

**Table 2:** Best-fit models (all *P* < 0.001, F-statistic) for accumulation of litter mixtures (leaves, twigs, bark) in eucalypt forests and woodlands at continental scale (X_Tsf_ (g m^2^) = I + *a*T_sf_ + *b*T_sf_^2^ + *c*AI + *d*Q_lf_)

Eucalypt forest/species	*n*	I	*a*	*b*	*c*	*d*	*R*^*2*^
All T_sf_							
All eucalypt forests	119	699 ^n.s.^	29.4 ***	−0.11 ***	1117 ***	-1303 **	0.44
Representative forests ^1^	85	862 ^n.s.^	35.7 ***	−0.13 ***	748 ^n.s.^	1107 ^n.s.^	0.45
T_sf_ <40 years							
All eucalypt forests	108	766 ^n.s.^	129***	−3.02***	1032***	-1806***	0.59
Representative forests ^1^	78	435 ^n.s.^	163***	−4.09***	984*	-1341*	0.65

^1^*E. regnans* data were excluded as there were too few observations to compute Q_lf_ (See also SI Table 1).

Independent variables (fixed effects) are elapsed time (since last fire, T_sf_, years), aridity index (AI) and quality of litterfall (Q_lf_). AI and Q_lf_ are defined in Methods. I = intercept, *a–d* are coefficients, *n* = number of replicates. For coefficients: *** = *P*<0.001, ** = *P*<0.01, * = *P* <0.05, n.s. = not significant (*P* > 0.05). All coefficient *P* values are based on t-statistics and two-sided test. As described in Methods, limited data for longer T_sf_ is a significant determinant of model performance. Models based on data for all eucalypt forests and all T_sf_ can be used for making predictions at continental scale. For comparative purposes, we include models where data is limited to (a) 40 years of elapsed time and (b) our Representative forests.

When AI and Q_lf_ were combined with T_sf_, modified quadratic functions were clearly superior to linear, exponential and power functions in describing litter accumulation (based on AIC and goodness-fit; *R*^2^). All models shown in Table 2 and Fig. 5 and Supplementary Tables 1–3 are highly significant (*P* < 0.001). We quantified improvements in models with stepwise inclusion of AI and Q_lf_ (Table 2, Supplementary Table 1). Alternative models based on polynoms (Supplementary Table 2) provide virtually identical results. As an alternative to Q_lf_, we also tested a measure of quality based on litter (Q_l_). While Q_lf_ relies on measurements of litterfall, Q_l_ relies on measurements of litter in situ and is thus influenced by rapid initial losses of mass and nutrients. Ql showed inferior results to Q_lf_ (e.g., Supplementary Table 3). Models for leaf litter were weaker than those for total litter (Supplementary Table 1).

As predicted from current decomposition theory, inclusion of AI and then Q_lf_ improved continental-scale models for both all eucalypt data and for our five representative forests (Table 2, Fig. 5, Supplementary Table 1). Addition of Q_lf_ (alongside AI) also improved ability of models to explain observed variance (Table 2, Fig. 5, cf Supplementary Table 1). As we note in Table 2, a continental scale model for eucalypts is logically best based on all available data. A model for our representative forests closely reflected the all-data model. With Q_lf_ as a fixed effect, a single model could account for ~45 % of the variance in litter mass for > 100 years for either our representative forests or all eucalypt communities across the entire continent (Table 2, Fig. 5, also Fig. 1). Model fit was improved by limiting the data to 40 years elapsed time (up to 65% explained variance). Models for individual forest communities could explain much of the variance in litter accumulation on the basis of elapsed time. For example, models for *E. marginata*, *E. diversicolor*, Grassy forests and Grassy woodlands explained most of the variance in litter mass (Supplementary Table 1) on the basis of elapsed time.

**Fig. 6:**
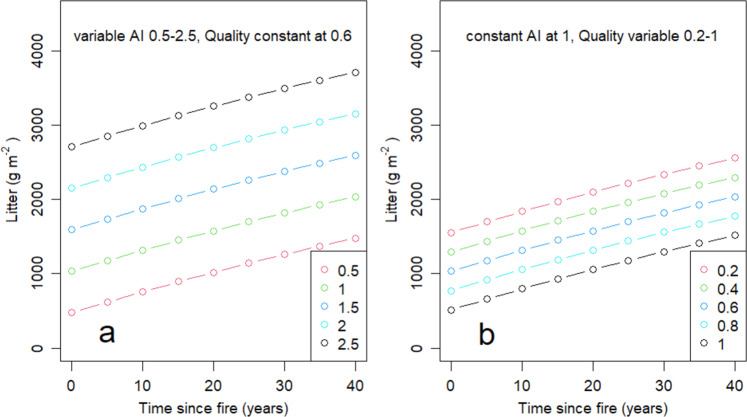
Model predictions of litter mass with increasing time. **a** Litter mass with fixed litterfall quality (Q_lf_) and a range of values for aridity index (AI), **b** Litter mass with fixed AI and a range of values of Q_lf_. Models represent all species from genera *Eucalyptus, Corymbia* and *Angophora*.

The robust generic model for complex litter layers in all eucalypt forests and woodlands (Table 2, Figs. 2, 5) can be represented in relation to climate and to input quality (Fig. 6). Figure 6A shows predictions for accumulation of litter over time if the quality of inputs (Q_lf_) is held constant. Figure 6B shows the same relationship for constant climates (AI). For comparison, Supplementary Figure 6 shows predictions based on Representative forests. These relationships are underpinned by the relationships of Q_lf_ to climate (Fig. 3) and X_Tsf_ to AI (Fig. 4).

## Discussion

This report provides a continental-scale analysis that meets the call for long-term studies of decomposition^26^. In doing so, it provides a robust test of understandings/hypotheses derived from thousands of shorter-term studies of decomposition, most often based on litterbags^8^. Climate and quality clearly regulate decomposition, and their effects are compounded through time.

As recognised in numerous reviews and meta-analyses – and notwithstanding important gains in knowledge - litterbag studies have limitations^3,8,13,15^. Combined with the data and analysis here, that recognition provides important caveats to past and future studies. First is that exponential behaviour should not be assumed in relation to decomposition of fallen litter in the field. It has been shown many times that rapid initial losses of mass from foliage are not sustained^17–19^, and such rapid initial losses are not in evidence for wood or bark. In fact, measured litter decomposition can be become so slow as to be incomplete, with portions of decomposing leaves accumulating for decades alongside wood and bark. Moreover, biotic regulators of decomposition (e.g. fungi and bacteria) are mostly non-specific ensuring that physical proximity of one tissue type to another (e.g. leaves, wood, bark) plays a role in buffering overall decomposition and accumulation of litter in forests. Fungal hyphae routinely ramify throughout the litter layer, colonise a wide range of substrates, and transfer carbon and nutrients within litter layers^33^ (also^6^ for the significance of fungi to distributions of tree species). Measures of quality of individual components are consequently only partially useful (also^7^) as guides to decomposition of litter layers. As we report (Supplementary Table 1), leaf litter accumulates at different rates to entire litter mixtures, and with a greater (unexplained) variation. Similarly, observed rates of decomposition of single species are seldom the same as rates of decomposition when foliage of two or more species are decomposing together^7,22,23^. While activities of decomposing soil fauna and microbial decomposers (fungi, bacteria etc) are often tightly interrelated^20,21^, physical proximity helps determine the biologically facilitated transfers of energy and nutrients, especially from more decomposable tissues to less decomposable tissues.

Secondly, use of decomposition constants (*k*) based on the assumption of exponential behaviour are highly misleading, and often wrong. *k* has been repeatedly shown to overestimate actual rates of decomposition, often drastically^34^, with the overestimation increasing in severity with litter age. The problems are compounded by incomplete decomposition^35–37^. Detailed studies have shown that, in effect, *k* varies continuously as decomposition proceeds, making highly questionable the pervasive use of single values of *k* for comparisons of tissue types, communities and of effects of geography.

Data and models presented here span a minimum 40-year period, or ~ 4 times the duration of the longest litter bag studies on record (e.g.^16–19^). Data for litterfall are more limited than those for litter (e.g. data available for calculating Q_lf_ is more limited than for Q_l_; Supplementary Table 3). Incorporated into regional-scale carbon models, functions presented in Fig. 6 can replace problematic use of *k* and significantly improve the accuracy and robustness of continental-scale estimates of carbon pools and fluxes. This outcome directly addresses the conclusions of Cornwell and Wheedon^38^: “progress will come from a model synthesis such that parameter estimates from empirical investigations using low-parameter models can be more clearly utilised in more complex ecosystem models”. We used a parsimonious approach and restricted our parameters to single measures of time, climate and quality.

Analysis of data for other continents are needed to confirm the findings reported here. Nonetheless, parsimonious models of litter accumulation show clear ability to explain variance in litter mass in situ for periods extending to >100 years. This may be contrasted with the longest-term (e.g. 10 years) and largest geographic-scale litterbag studies^10,16–19^ where, despite large numbers of independent variables, explained variance in remaining foliage rarely rises above 60% for individual sites and is often considerably less across sites. Robust predictions of the mass of litter in forests will require more studies such as that reported here.

The inclusion of AI resulted in significant improvement in explained variance at both the community and continental scale. This is perhaps expected since moisture and temperature are the best-known of all abiotic drivers of decomposition. Less expected were gains in predictive power due to the inclusion of a measure of overall litter quality (Q_lf_). At the continental scale, Q_lf_ shows a strong dependence on climate, that is not evident at local (within the community) scales. As shown in Fig. 4, the climate-Q_lf_ relationship has the hallmarks of an emergent ecosystem property. The ecological significance of emergent properties has a long history (e.g.^39^; also^10^ for discussion in relation to decomposition). The indirect effect of climate on decomposition reported here might be assessed in relation to its better-known, direct effects. The latter are mostly scale-invariant while the indirect effects of climate on the decomposition of complex mixtures via input quality show scale-dependence (*sensu*^10^).

After these direct and indirect climate effects on litter accumulation, a third, and highly distinctive role of climate lies in its undisputed control of geographic extent, biotic composition and productivity of plant communities generally^40^, including the hundreds of distinct, eucalypt-dominated communities that vary widely in accumulated litter mass (e.g. Figures 1, 2, Table 2). Climate control of distributions of biota and their activity is an enduring topic in ecology.

For the Australian continent (noting there are >800 species of *Eucalyptus, Corymbia, Angophora*), climatic control is expressed in emphatic differences in rates of accumulation among distinct eucalypt-dominated communities. Differences in litter accumulation between forest types that are geographically close yet clearly different in climate and productivity, such as between Jarrah (*E. marginata*) and Karri (*E. diversicolor)* forests of WA (Fig. 2), or between the Mountain Ash (*E. regnans*) and Messmate (*E. obliqua*) forests of south-eastern Australia (including Tasmania, e.g.^32^), are so large as to be self-evident. At the same time, more subtle climate controls, for example the biotic control of the quality of input mixtures (i.e. Q_lf_;), have not hitherto been considered at the community level. As we show, inclusion of Q_lf_ as a fixed effect (independent variable) improved ability to predict decomposition and thus litter accumulation (Table 2, Fig. 5, Supplementary Table 1). Increased shedding of leaves in response to drought is a well-known phenomenon across all global forests and will increase Q_lf_. As droughts intensify leaf shedding extends to become canopy dieback and eventually tree mortality^41^, with concomitant increases in quantities of twigs and bark in litterfall. In a rare, long-run study of eucalypt litterfall, stochasticity in shedding of more woody materials was clearly greater than that of leaves and flowers/fruits^42^ and all eucalypt forests show strong year-to-year variation in litterfall (e.g. Fig. 1). Similar stochasticity appears as site- (or scale-) dependent behaviour in the biotic communities represented in Fig. 4 and underpins the large range in Q_lf_ for a given AI. Climate can also mask and/or amplify biotic effects. For example, termites are important decomposers of woody materials^43^, and their abundance follows bioclimatic distributions being clearly greater in warm-hot northern and inland Australia. Termites thus contribute to the predictability of litter accumulation in Grassy forests and woodlands (Supplementary Table 1). Similarly, ground-dwelling birds that churn the litter in their search for food (e.g. Superb Lyrebird, *Menura novaehollandiae*) may have significant effects on leaf decomposition within *E. regnans* forests^44^ (also Ash forest model, Supplementary Table 1). However, these birds are not found outside the cool, wet forests of SE Australia. Assignment of proximal biotic drivers (i.e. region- or forest-specific) will nearly always require a caveat of careful consideration of a priori (albeit more distal) effects of climate on biotic distributions, abundances and activities (e.g. forest productivity).

In addition to avoiding an a priori assumption that litter decomposition and accumulation should show exponential behaviour (see above), we also avoided assuming a limit to litter mass. However, both assumptions are widely used in fire risk modelling^29^. They are also used in policy and practise by land management and fire-fighting agencies in Australia (Supplementary Table 4). These assumptions ignore published studies showing that in climate conditions strongly favourable to decomposition, eucalypt forests still accumulate litter for many decades^45,46^, even centuries^47^. One recent study^48^ attributed the increased extent of forest fires to changing climates using model predictions that fine litter and very fine litter were constant through time based on assumed limits. Similarly, Bowman et al.^49^ concluded that the carbon costs of prescribed fire (used as a management tool to aid fire suppression) outweighed its possible benefits, based again on model assumptions that fine fuels quickly reach a limit after the fire. Results here suggest neither assumption holds within the range of the data. Litter accumulates continuously for at least 40 years. Drying climates will change fine fuel loads (and most likely fire extent and severity) unless offset by climate-driven changes to litter quality. Assumptions of exponential behaviour of litter accumulation, and especially of limits, are not supported by the data and fire risk models need to be adjusted accordingly.

At longer time scales, the balance of the two independent and opposing processes that drive litter accumulation – litterfall and litter decomposition – will continue to determine litter mass. Water retention within the litter layer and especially the prevention of evaporation from underlying soil via the insulative properties of litter, increase with time. These mulching effects are well described in the literature (classic paper from MacKinney^50^, also^51,52^) and promote decomposition especially during dry (summer) periods in water-limited forests. Decomposition losses may thus eventually exceed annual litterfall inputs such that overall litter mass declines. This is reflected in the modestly quadratic character of the model(s) reported here. There is no biological basis for assuming the two processes reach and stay in equilibrium.

In contrast to conclusions that litter responses to rising CO_2_ would be limited to a few years^53^, analysis here suggests that consideration of non-foliage components of litter and litterfall greatly extend the temporal extent of effects of rising CO_2_ on the accumulation of carbon in complex litter layers. Similarly, climate change-driven differences in crown dieback and tree decline among functional groups, genera and species^54^ must eventually be reflected in longer-term impacts on decomposition, litter accumulation and fire risk. Giving priority to long-term studies of litter accumulation^26^ should be extended to the quality and quantity of inputs (litterfall). Climatic controls are both direct via control of physio-chemical and biological activity, and indirect via inputs that vary in quantity and quality (also^55^) yet remain overlooked in models of carbon in forests globally. Droughts are significant causes of canopy dieback and must eventually increase all of leaf, twig, and bark litterfall, with ensuing effects for decomposition, litter accumulation and fire risk. The lignin bottleneck^56^ – expressed in control of decomposition by species identity and tissue type – will remain subject to direct and indirect moderation by climate^6^.

## Methods

### Data

Continental datasets on litter and litterfall based on destructive sampling are scarce in comparison to similar scale data on forest biomass and structure that are often collected for national forest inventories^57,58^. Example data sets include those from the ICP program which includes assessments of organic soil layers and litter stocks on the forest floor for about 4900 plots, as well as annual litterfall for about 320 plots^1,59^. The United States forest inventory (FIA) includes data for litter and coarse woody debris for ~5000 field plots^60,61^. We are not aware of similar datasets on litterfall and litter for Africa. The most recent global compilation of litter stocks and litterfall was prepared by Holland et al.^62^ who used the work of Bray and Gorham^63^ as a basis.

### Eucalyptus litter and litterfall

**Fig. 7:**
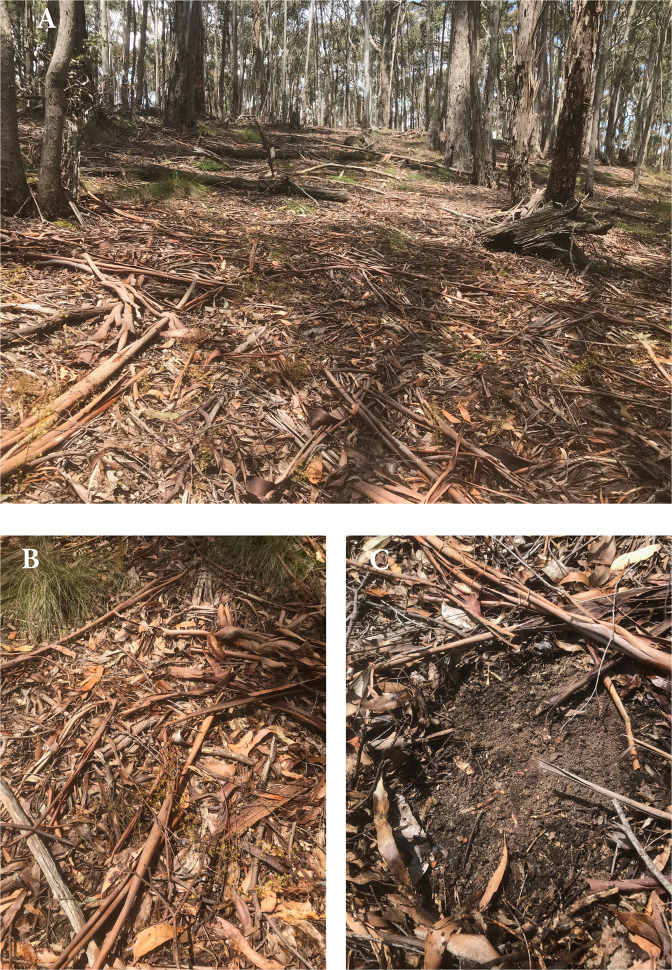
Litter accumulation in long unburnt Wombat Forest, near Daylesford, Vic. **A** This forest is dominated by *Eucalyptus rubida* (Candlebark) along with *E. radiata* (Narrow leaf peppermint). **B** Shed bark and twigs decompose much more slowly than foliage. **C** Litter sampling is aided by distinct boundaries from litter (Oi+Oe) to humified material (Oa), and from Oa to mineral soil.

Litter layers in eucalypt forests and woodlands (Fig. 7) differ from many Northern Hemisphere coniferous or hardwood forests where dead foliage is the dominant component over centuries. Eucalypt litter layers shift from foliage-domination in the first few years after fire, to being skewed toward more lignified components such as twigs and bark while still including lignified parts of foliage (e.g. Fig. 7B). In addition, eucalypt forests and woodlands do not fit neatly into ‘humus’ classifications developed for northern hemisphere forests^64,65^. For example, organic matter deposited and decomposing on the forest floor in eucalypt forests can generally be recognised as either Oi or Oe with a distinct (and generally very thin) Oa layer below. However, further sub-classification of humus forms is rare for Australian forests. We have used here a definition of ‘litter’ that includes Oi and Oe fractions but excludes Oa. A few other general features of forest floors are noteworthy. Mull humus forms are largely absent from eucalypt forests and woodlands. In common with nutrient-poor conifer forests, Mor humus is by far the most common in eucalypt forests and woodlands, with sharp/clear boundaries between organic and mineral soil horizons (e.g. Fig. 7C). Sharp/clear boundaries between mineral soil and Oa horizons, and between Oa and Oe horizons, greatly facilitate litter sampling. Moder humus is found in some of the more productive forests where birds and other biota churn the litter with soil. Some authors have used sieves (e.g. 2 mm) to separate Oi + Oe (decomposing leaves, twigs, bark and fragments thereof) from more clearly humified material of smaller particle size (Oa), but generally litter sampling is based on visual recognition of identifiable plant components and fragments thereof. Charcoal is routinely excluded from litter sampling.

### Eucalyptus communities and fire

In temperate coniferous and broadleaf forests of Europe and North America, single species frequently dominate over large areas with simple or even absent understories. In contrast, eucalypt forests and woodlands: (i) are highly diverse in dominant species, (ii) have diverse and complex understories (both herbaceous and woody) and frequently distinct overall compositions of flora and fauna species, and (iii) range greatly in productivity and many other parameters. In practise, many species of *Eucalyptus* or *Corymbia* or *Angophora* (or eucalypts *sensu lato*) cover sufficiently large areas to be regarded as the dominant species of distinct biotic communities. Much of Australia’s eucalypt-dominated forest and woodland burns at moderate-high frequencies^66,67^. These ecosystems span a 30^o^ range in latitude, 40^o^ in longitude, and a large proportion lies within the globally dry latitudes of 20^o^ to 30^o^S. Nearly all eucalypt ecosystems - including those furthest south – can be hot and dry for significant periods most years owing to topography and geographic location. Eucalypt forests and woodlands share many characteristics that increase or promote flammability across the 800+ species in the three genera - *Eucalyptus, Corymbia* and *Angophora*^66^. Frequent fires thus provide opportunities to directly quantify the accumulation of litter over following years and decades. Most moderate-high intensity fires in these ecosystems consume the litter layers, leaving humified material, charcoal and ash. Low intensity fires are inherently patchier, leaving unburnt litter in some places but consuming the litter in others. Frequent thus provide opportunities to directly quantify the accumulation of litter over following years and decades.

We collated the data used here and have lodged it in a publicly accessible storage. The full data set contains more than 1812 individual datapoints for litterfall and 3887 datapoints for standing litter, from the Australian continent. We limited the present analysis to eucalypt forests and woodlands within the database (Fig. 1). Notable exclusions are: (1) areas of wet tropical forests (where canopy dominance is frequently shared across a range of genera and species), (2) extensive inland areas of shrubland dominated by *Acacia* spp, (3) woodlands dominated by *Casuarina* spp. and *Callitris* spp., (4) softwood and hardwood plantations. For all eucalypt sites, the database includes the dominant canopy species as well as a range of geographic and climate details and fire records.

### Litter sampling

Litter and litterfall are typically sorted into categories of plant parts prior to weighing – leaves, wood, fruits, bark. Wood and bark data thus received additional attention owing to some inconsistencies in reporting. Wood, and sometimes bark, in litterfall and litter studies was nearly always further classified according to particle size (often diameter). Some 73% of all wood and bark observations in the database used a 6 mm thickness or diameter limit to distinguish litter and litterfall from branchfall and tree mortality. Material of larger physical dimensions is often classed as branchwood (or even stemwood with attached bark) and is routinely excluded from litterfall analyses. A further 7% of observations are based on a 10 mm limit, while a further 20% used 26 mm. We used observations of mass of components of litter and litterfall as they were reported (i.e. including variation in size limits of twigs). While this approach introduces additional small sources of variation, we considered it superior to and more transparent than applying correction factors to standardise data to a specific size limit.

### Data analysis

The data set contains > 274 measurements for C and N concentrations (leaves = 194, wood+bark = 61, other = 19) for eucalypt forests and woodlands. C:N ratios of woody material (twigs and bark) are at least 50% greater than C:N ratios of leaves, in both litter and litterfall (Supplementary Fig. 3). Other materials (mainly fruiting structures, leaf fragments) have intermediate C:N. Other measures of quality (e.g. lignin concentration) had too few observations to be used reliably.

The data set for litter is biased with respect to time since fire (T_sf_, Supplementary Fig. 5). T_sf_ was recorded for around half of all 3800 litter observations and > 75% of those fell within 20 years, with 92% for Tsf < 40 years. We thus analysed the data on the basis of (a) all observations and (b) observations limited to 40 years T_sf_ (e.g. Table 2, Fig. 5, Supplementary Tables 1–3). Assignment of T_sf_ becomes increasingly difficult beyond 40 years T_sf_ owing to the nature of publicly available fire records (especially fire extent and severity). Relative to the number of observations, such data for long T_sf_ may have outsize influence in statistical analyses.

For five eucalypt communities (as defined by dominant eucalypt species) shown in Fig. 1, there is sufficient data to allow robust analysis and these five comprise 40% of all available litter data (Table 1). An alternative to our use of dominant eucalypt to define communities is to aggregate data into groups via classification or other means. An example classification is ‘vegetation formations’ based on vegetation structure (e.g.^68^; Supplementary Fig. 2). Another classification is ‘bioclimatic zones’ (e.g.^69^), while a third, often-used grouping is based on genetics of the dominant eucalypts (e.g.^70^). No aggregation offers a perfect solution. Structural classifications do not explicitly account for large variations in biotic composition, productivity and climate. Bioclimatic zones can range in extent up to thousands of kilometres, again spanning several-fold variations in productivity, litterfall and litter. Equally, genetic similarity does not ensure similarity in terms of productivity or structure. Aggregation can result in extreme ranges in litter data with many outliers (Supplementary Fig. 2). Using the species identity of the dominant eucalypt as the primary proxy for each biotic community helps avoid some of the inherent problems in data aggregation^9,10^. Eucalypt species interchange as dominants at scales as small as tens to hundreds of metres across sometimes almost imperceptible variations in topography, soils and micro-climate^71^.

### Models and modelling

We used multivariate models to quantify the importance of T_sf_, quality (Q), and Aridity Index (AI) on litter accumulation (X_Tsf_). We used T_sf_ for each datapoint from the literature while Aridity Index (Precipitation: Potential Evapotranspiration) includes the influences of M (moisture) and T (temperature). We tested a range of model functions (linear, power, exponential, polynomial) and a range of measures of climate (e.g. precipitation, AI, climate scalar^31,32^). We report here best-fit models; tabulated analyses for other models and climate measures are available on request from the authors.

When treated as a whole, the quality (Q) of litter with respect to decomposition depends on relative proportions of more decomposable leaf material (lesser C:N) and much slower-to-decompose wood/bark (greater C:N). We thus tested two proxy metrics of overall quality of litter for decomposition. One metric (Q_l_) is the ratio of (mass of leaf litter): (mass of total litter). A second metric (Q_lf_) is the (mass of leaf litterfall): (mass of total litterfall). Q_l_ reflects both litterfall inputs and their decomposition and is less directly related to initial quality of litter for decomposition and less easily interpreted than Q_lf_ (Supplementary Table 3). Inclusion of Q_lf_ strongly increased explained variance in multivariate models (compare Fig. 5, Supplementary Table 1). Q_lf_ was moderately superior to Q_l_ in terms of improving ability to explain variance in X_Tsf_ (compare Table 2, Fig. 5, Supplementary Table 3). Simple quadratic models can account for most of the variance in litter mass within individual forest communities or formations. As expected, time is clearly the dominant driver and most likely accounts for the quadratic aspects of the models (e.g., Fig. 6).

On-going accumulation of litter with time modifies litter-soil micro-climates, insulating soil and deeper litter layers (also^72^). In particular, lower litter layers and soils are protected from evaporation. The afforded protection helps maintain moisture status and decomposition activity during summer in typically water-limited eucalypt forests and woodlands and is a powerful feedback to rates of decomposition. These effects increase with time. Consequently, annual losses of mass through decomposition may eventually exceed annual inputs from litterfall, and litter mass may eventually thus decline.

### Statistics

We reported heteroscedasticity-consistent errors using “lm_robust” function of *R* statistical software^73^. We used coefficient errors and coefficients of multiple determination (*R*^*2*^) and Akaike’s Information Criterion (AIC) to compare model performance. As reported (Fig. 2, Supplementary Table 1), our statistical analysis includes multicollinearity. We addressed the collinearity issue using orthogonal polynoms (Supplementary Table 2) and found no difference in overall proportions of explained variance (*cf* Fig. 2; Supplementary Table 1). We have presented models based on raw polynomials for general ease of understanding and interpretation. Robust comparisons of covariate importance might be better based on orthogonal polynoms (Supplementary Table 2). We fitted multivariate models using both total litter (i.e., litter mixtures including leaves, fruits and woody material) and leaf litter as target variables.

Statistical analyses (visualisation, regression analysis) were prepared using R statistical software^73^. We assigned vegetation formations according to location and determined the dominant tree species in the overstory from the data references. We complemented litterfall and litter data with C and N measurements made on the same sites. Climate data used in calculation of Aridity Indices represent the period 1970-2000 and were extracted from TerraClimate data base^74^.

### Reporting summary

Further information on research design is available in the Nature Portfolio Reporting Summary linked to this article.

## Supplementary information

Supplementary Information

Reporting Summary

## Data Availability

The litter and litterfall data generated in this study have been deposited in the Figshare database under accession code 13160351.
