# Author Correction: Litter accumulation and fire risks show direct and indirect climate-dependence at continental scale

**DOI:** 10.1038/s41467-024-50152-z

**Published:** 2024-08-01

**Authors:** Mark A. Adams, Mathias Neumann

**Affiliations:** 1 School of Science, Computing and Engineering Technologies, Swinburne University of Technology, Hawthorn, VIC Australia; 2 Institute of Silviculture, Department of Forest and Soil Sciences, University of Natural Resources and Life Sciences, Vienna, Austria

**Keywords:** Carbon cycle, Natural hazards, Ecosystem ecology

Correction to: *Nature Communications* 10.1038/s41467-023-37166-9, published online 18 March 2023

The original version of this Article contained an error in Table 2 and Figure 5, in which models of litter accumulation were included at continental scale that incorporated effects of quality and climate – but wrongly also included models of the same structure for individual forest communities. This has been corrected in both the PDF and HTML versions of the Article.

The original version of the Supplementary Information associated with this Article contained an error in SI tables 1 and 2, which did not include best-fit quadratic models for individual forest types, that rely solely on time. The correct version states now includes said models, plus models for aggregated forest types, where aggregation was based on either forest structure or genetic similarity. The HTML has been updated to include a corrected version of the Supplementary Information.

The original version of this Article contained an error in the Abstract, which incorrectly read *‘Using a data set for the Australian continent, we show that among a range of models, >60% of the variance in litter mass over a 40-year time span can be accounted for by a parsimonious model with elapsed time, and indices of aridity and litter quality, as independent drivers. Aridity is an important driver of variation across large geographic and climatic ranges while litter quality shows emergent properties of climate-dependence. Up to 90% of variance in litter mass for individual forest types can be explained using models of identical structure.’* **The correct version states** *‘Using a data set for the Australian continent, we show that among a range of models, most of the variance in litter mass over a 40-year time span can be accounted for by a parsimonious model with elapsed time, and indices of climate and litter quality, as independent drivers. Climate is an important driver of variation in both the species identity of dominant eucalypts and litter accumulation across the continent. Litter quality shows emergent properties of climate-dependence and contributes to explained variance. Nonetheless, elapsed time dominated explained variance in litter mass (up to 90%) at continental scale.’* This has been corrected in both the PDF and HTML versions of the Article.

The original version of this Article contained an error in Introduction section which incorrectly read ‘*Importantly, accumulated litter is also a driver of wildfires owing to its mass, its ease of ignition and combustion under dry conditions, and the strong relationship of fire intensity to consumed fuel24’.* **The correct version states** ‘*Importantly, accumulated litter (< 6mm diam.; 'fine fuel') is also a driver of wildfires owing to its mass, its ease of ignition and combustion under dry conditions, and the strong relationship of fire intensity to consumed fuel24*’. This has been corrected in both the PDF and HTML versions of the Article.

The original version of this Article contained an error in the Results section, Characteristics of litter and litterfall, which incorrectly read ‘*We also aggregated data for two vegetation formations of large geographic extent (Grassy forests, Grassy woodlands; Table 1: Supplementary Table 1: Supplementary Fig 2), and separately for ‘Ash forests’ based on genetic similarity (data available for = E. regnans, E. delegatensis, E. sieberi), thereby including a further 30% of all litter data.*’ **The correct version states** *‘We also aggregated data for two vegetation formations of large geographic extent (Grassy forests, Grassy woodlands; Table 1: Supplementary Table* *1**: Supplementary Fig* *2**), and separately for ‘Ash forests’ based on genetic similarity (data available for Ash forests = E. regnans, E. delegatensis, E. sieberi) - a further 30% of all litter data.’*. This has been corrected in both the PDF and HTML versions of the Article.

The original version of this Article contained an error in the Results section, Modelling litter accumulation, which incorrectly read ‘*While Q*_*lf*_ *relies on measurements of litterfall, Q*_*l*_ *relies on measurements of litter in situ and is thus influenced by rapid initial losses of mass and nutrients. Q*_*l*_ *showed strongly inferior results to Q*_*lf*_ *(Supplementary Table* *3). Models for leaf litter were weaker than those for total litter (Supplementary Table* *1)*.

*As predicted from current decomposition theory, inclusion of AI and then Q*_*lf*_ *improved all models; those for individual communities, a generic model for all eucalypts, and models based on aggregated data (Methods) for Grassy woodlands, Grassy forests and Ash forests (Table* *2, Fig*. *5, Supplementary Table* *1). Inclusion of AI as a fixed effect increased explained variance in every community, in every aggregation, and when all eucalypt forests and woodlands were considered together (Supplementary Table* *1). More profoundly, addition of Q*_*lf*_ *(alongside AI) led to a strong improvement in ability of models to explain observed variance (Table* *2, Fig*. *5, cf* *Supplementary Table* *1). When Q*_*lf*_ *is included as a fixed effect, a single model could account for >60% of the variance in litter mass over 40 years for all eucalypt communities across the entire continent (Table* *2, Fig*. *5, also Fig*. *1). Models for individual communities could in some instances explain almost all the variance in litter accumulation. For example, models for E. marginata, Grassy forests and Grassy woodlands explained almost all the variance (>90%) in litter mass (Table* *2, Fig*. *5). Models for E. diversicolor and Ash forests (aggregated data) had only slightly less explanatory power (80%+)*.

*The robust generic model for complex litter layers in eucalypt forests and woodlands (Table* *2, Figs*. *2* *and* *5) can be represented in relation to climate and to input quality (Fig*. *6). Figure* *6A* *shows predictions for accumulation of litter over time if the quality of inputs (Q*_*lf*_*) is held constant. Figure* *6B* *shows the same relationship for constant climates (AI). These relationships are underpinned by the relationships of Q*_*lf*_ *to climate (Fig*. *3) and X*_*Tsf*_ *to AI (Fig*. *4)*.’ **The correct version states** ‘*While Q*_*lf*_ *relies on measurements of litterfall, Q*_*l*_ *relies on measurements of litter in situ and is thus influenced by rapid initial losses of mass and nutrients. Q*_*l*_ *showed inferior results to Q*_*lf*_ *(e.g*., *Supplementary Table* *3**). Models for leaf litter were weaker than those for total litter (Supplementary Table* *1**)*.

*As predicted from current decomposition theory, inclusion of AI and then Q*_*lf*_ *improved continental-scale models for both all eucalypt data and for our five representative forests (Table 2, Fig. 5*, *Supplementary Table* *1**). Addition of Q*_*lf*_ *(alongside AI) also improved ability of models to explain observed variance (Table 2, Fig. 5, cf* *Supplementary Table* *1**). As we note in Table 2, a continental scale model for eucalypts is logically best based on all available data. A model for our representative forests closely reflected the all-data model. With Q*_*lf*_ *as a fixed effect, a single model could account for ~45 % of the variance in litter mass for > 100 years for either our representative forests or all eucalypt communities across the entire continent (Table 2, Fig. 5, also Fig. 1). Model fit was improved by limiting the data to 40 years elapsed time (up to 65% explained variance). Models for individual forest communities could explain much of the variance in litter accumulation on the basis of elapsed time. For example, models for E. marginata, E. diversicolor, Grassy forests and Grassy woodlands explained most of the variance in litter mass (Supplementary Table* *1**) on the basis of elapsed time*.

*The robust generic model for complex litter layers in all eucalypt forests and woodlands (Table 2, Figs. 2, 5) can be represented in relation to climate and to input quality (Fig. 6). Figure 6A shows predictions for accumulation of litter over time if the quality of inputs (Q*_*lf*_*) is held constant. Figure 6B shows the same relationship for constant climates (AI). For comparison*, *Supplementary Fig.* *6**shows predictions based on Representative forests. These relationships are underpinned by the relationships of Q*_*lf*_ *to climate (Fig. 3) and X*_*Tsf*_ *to AI (Fig. 4)*.’ This has been corrected in both the PDF and HTML versions of the Article.

The original version of this Article contained an error in the Discussion section, which incorrectly read ‘*Data and models presented here span a 40-year period, or ~ 4 times the duration of the longest litter bag studies on record (e.g*. ^*16–19*^).’ **The correct version states** ‘*Data and models presented here span a minimum 40-year period, or ~ 4 times the duration of the longest litter bag studies on record (e.g*. ^*16–19*^*)*’. This has been corrected in both the PDF and HTML versions of the Article.

The original version of this Article contained an error in the Discussion section, which incorrectly read ‘*Analysis of data for other continents are needed to confirm the findings reported here. Nonetheless, parsimonious models of litter accumulation – for forests and woodlands dominated by single eucalypt species, for aggregated forests and woodlands, and for all eucalypt forests and woodlands – show clear ability to explain variance in litter mass in situ (i.e. R*^*2*^ *range from 60% to >95%). This may be contrasted with the longest-term and largest geographic-scale litterbag studies*^*10,16–19*^ *where, despite large numbers of independent variables, explained variance in remaining foliage rarely rises above 60% and is often considerably less. Robust predictions of the mass of litter in forests will require more studies such as that reported here*.’ **The correct version states** ‘*Analysis of data for other continents are needed to confirm the findings reported here. Nonetheless, parsimonious models of litter accumulation show clear ability to explain variance in litter mass in situ for periods extending to >100 years. This may be contrasted with the longest-term (e.g. 10 years) and largest geographic-scale litterbag studies*^*10,16–19*^ *where, despite large numbers of independent variables, explained variance in remaining foliage rarely rises above 60% for individual sites and is often considerably less across sites. Robust predictions of the mass of litter in forests will require more studies such as that reported here*’. This has been corrected in both the PDF and HTML versions of the Article.

The original version of this Article contained an error in the Discussion section, which incorrectly read ‘*Termites thus contribute to the exceptional predictability of litter accumulation in Grassy forests and woodlands (Table* *2, Fig*. *5, Supplementary Table* *1). Similarly, ground-dwelling birds that churn the litter in their search for food (e.g. Superb Lyrebird, Menura novaehollandiae) may have significant effects on leaf decomposition within E. regnans forests*^*44*^ *(also Ash forest model, Table* *2, Fig*. *5). However, these birds are not found outside the cool, wet forests of SE Australia. In both examples, much of the variance in accumulated litter mass in both example ecosystems could be accounted for by elapsed time, litter quality and climate. Assignment of proximal biotic drivers (i.e. region- or forest-specific) will nearly always carry a caveat of careful consideration of a priori (albeit more distal) effects of climate on biotic distributions, abundances and activities (e.g. forest productivity)*.’ **The correct version states** ‘*Termites thus contribute to the predictability of litter accumulation in Grassy forests and woodlands (Supplementary Table* *1**). Similarly, ground-dwelling birds that churn the litter in their search for food (e.g. Superb Lyrebird, Menura novaehollandiae) may have significant effects on leaf decomposition within E. regnans forests*^*44*^ *(also Ash forest model*, *Supplementary Table **1**). However, these birds are not found outside the cool, wet forests of SE Australia. Assignment of proximal biotic drivers (i.e. region- or forest-specific) will nearly always require a caveat of careful consideration of a priori (albeit more distal) effects of climate on biotic distributions, abundances and activities (e.g. forest productivity)*’. This has been corrected in both the PDF and HTML versions of the Article.

The original version of this Article contained an error in the Methods section, Eucalyptus communities and fire, which incorrectly read ‘*Eucalypt forests and woodlands share many characteristics that increase or promote flammability across the 800+ species in the three genera - Eucalyptus, Corymbia and Angophora66. Most moderate-high intensity fires in these ecosystems consume the litter layers, leaving humified material, charcoal and ash*.’ **The correct version states** ‘*Eucalypt forests and woodlands share many characteristics that increase or promote flammability across the 800+ species in the three genera - Eucalyptus, Corymbia and Angophora66. Frequent fires thus provide opportunities to directly quantify the accumulation of litter over following years and decades. Most moderate-high intensity fires in these ecosystems consume the litter layers, leaving humified material, charcoal and ash*.’ This has been corrected in both the PDF and HTML versions of the Article.

The original version of this Article contained an error in the Methods section, Eucalyptus communities and fire, which incorrectly read ‘*Notable exclusions are: (1) areas of wet tropical forests (where canopy dominance is frequently shared across a range of genera and species), (2) extensive inland areas of shrubland dominated by Acacia spp, (3) woodlands dominated by Casuarina and Callitris, (4) softwood and hardwood plantations. For all eucalypt sites, the database includes the dominant canopy species as well as a range of geographic and climate details and fire records*.’ **The correct version states** ‘*Notable exclusions are: (1) areas of wet tropical forests (where canopy dominance is frequently shared across a range of genera and species), (2) extensive inland areas of shrubland dominated by Acacia spp, (3) woodlands dominated by Casuarina spp. and Callitris spp., (4) softwood and hardwood plantations. For all eucalypt sites, the database includes the dominant canopy species as well as a range of geographic and climate details and fire records*. ’. This has been corrected in both the PDF and HTML versions of the Article.

The original version of this Article contained an error in the Methods section, litter sampling, which incorrectly read ‘*Litter and litterfall are typically sorted into categories of plant parts prior to weighing – leaves, wood, fruits, bark, and the database reflects this. Wood and bark data received additional attention owing to some inconsistencies in reporting*.’ **The correct version states** ‘*Litter and litterfall are typically sorted into categories of plant parts prior to weighing – leaves, wood, fruits, bark. Wood and bark data thus received additional attention owing to some inconsistencies in reporting*.’ This has been corrected in both the PDF and HTML versions of the Article.

The original version of this Article contained an error in the Methods section, data analysis, which incorrectly read ‘*The data set for litter is biased with respect to time since fire (T*_*sf*_*, Supplementary Fig*. *5). T*_*sf*_ *was recorded for around half of all 3800 litter observations and > 75% of those fell within 20 years, with 92% for T*_*sf*_ *<* *40 years. We used a limit of 40 years T*_*sf*_ *for most analyses (e.g. Table* *2, Figs*. *5* *and* *6, Supplementary Tables* *1–3). Assignment of T*_*sf*_ *becomes increasingly arbitrary beyond this limit owing to the nature of publicly available fire records (especially fire extent and severity). The few data points for long T*_*sf*_ *(e.g. 40+ years) could also have undue influence if included in statistical analyses. Forty-year records of litter accumulation for a range of forest types at a continental scale are rare*.’ **The correct version states** ‘*The data set for litter is biased with respect to time since fire (T*_*sf*_, *Supplementary Fig. **5**). T*_*sf*_ *was recorded for around half of all 3800 litter observations and > 75% of those fell within 20 years, with 92% for T*_*sf*_ *<40 years. We thus analysed the data on the basis of (a) all observations and (b) observations limited to 40 years T*_*sf*_ *(e.g. Table 2, Fig. 5*, *Supplementary Tables* *1**–**3**). Assignment of T*_*sf*_ *becomes increasingly difficult beyond 40 years T*_*sf*_ *owing to the nature of publicly available fire records (especially fire extent and severity). Relative to the number of observations, such data for long T*_*sf*_ *may have outsize influence in statistical analyses*’. This has been corrected in both the PDF and HTML versions of the Article.

The original version of this Article contained an error in the Methods section, models and modelling, which incorrectly read ‘*Q*_*lf*_ *was clearly superior to Q*_*l*_ *in terms of improving ability to explain variance in XT*_*sf*_ *(compare Table 2, Fig. 5, Supplementary Table 3). Q*_*lf*_ *has a more limited data set (reduced observations) but when included in best-fit models as a fixed effect (independent variable) resulted in 80%+ explained variance in litter mass for Grassy forest, Grassy woodland and Ash forest aggregations, as well as for E. marginata and E. diversicolor forests (Table 2, Fig. 5). For all eucalypt forests and woodlands across the Australian continent, the best-fit, modified quadratic model includingQ*_*lf*_ *accounted for 60% of the variance in litter mass, while a best-fit model for selected representative forests accounted for 65% of the variance (Table 2, Fig. 5). Models of the same structure could account for 90%+ of variance in litter mass within individual forest types or formations. Time is clearly the dominant driver and most likely accounts for the quadratic aspects of models (e.g. Fig. 6). On-going accumulation of litter with time modifies litter-soil micro-climates, insulating soil and deeper litter layers (also*^*72*^*). The afforded protection helps maintain moisture status and decomposition activity during summer in typically water-limited eucalypt forests and woodlands. These effects become more pronounced at longer time scales (e.g. 40–250 years), when annual losses of mass through decomposition may exceed annual inputs from litterfall, and litter mass may thus decline.’* **The correct version states** ‘*Q*_*lf*_ *was moderately superior to Ql in terms of improving ability to explain variance in XT*_*sf*_ *(compare Table 2, Fig. 5, Supplementary Table 3). Simple quadratic models can account for most of the variance in litter mass within individual forest communities or formations. As expected, time is clearly the dominant driver and most likely accounts for the quadratic aspects of the models (e.g., Fig. 6). On-going accumulation of litter with time modifies litter-soil micro-climates, insulating soil and deeper litter layers (also*^*72*^*). In particular, lower litter layers and soils are protected from evaporation. The afforded protection helps maintain moisture status and decomposition activity during summer in typically water-limited eucalypt forests and woodlands and is a powerful feedback to rates of decomposition. These effects increase with time. Consequently, annual losses of mass through decomposition may eventually exceed annual inputs from litterfall, and litter mass may thus decline.’* This has been corrected in both the PDF and HTML versions of the Article.

### Supplementary information

Updated Supplementary Information

