# Supplementary material for: Litter accumulation and fire risks show direct and indirect climate-dependence at continental scale
Source: Nat Commun. 2023 Mar 18;14:1515. doi: 10.1038/s41467-023-37166-9 (PMC10024763; doi:10.1038/s41467-023-37166-9)
Supplement: Supplementary file 1 — Supplementary Information [file 41467_2023_37166_MOESM1_ESM.pdf]

Supplementary Information

Litter accumulation and fire risks show direct and indirect climate-dependence at continental scale.

Authors: Mark A. Adams[1*], Mathias Neumann[1,2]

Affiliations:

[1]School of Science, Computing and Engineering Technologies, Swinburne University of Technology, Hawthorn, Victoria, Australia.

[2]Institute of Silviculture, Department of Forest and Soil Sciences, University of Natural Resources and Life Sciences, Vienna, Austria.

*Corresponding author. Email: maadams@swin.edu.au

Supplementary Table 1

Coefficients, significance and coefficient of determination (R^2) for best-fit models (all models P<0.001, F-statistic) of litter accumulation (X_{Tsf}, g m^{-2}) in eucalypt communities. Independent variables are time since last fire (T_{sf}, years), aridity index (AI) and quality of litterfall inputs (Q_{lf}). I = intercept, *a-d* are coefficients, *n* = number of observations. Also shown are models for individual forest types as well as aggregated forest types (see Methods). For coefficients: *** = P < 0.001, ** = P < 0.01, * = P < 0.05, ^{ns} = not significant (P>0.05). All coefficient P values are based on t-statistics and two-sided tests.

	Model for X_{Tsf}	*n*	I	*a*	*b*	*c*	*d*	R^2
				All litter, all Tsf				
All eucalypt forests[1]	$I + aT_{sf} + bT_{sf}^2$	1715	696 ***	38.8 ***	-0.14 ***	-	-	0.266
	$I + aT_{sf} + bT_{sf}^2 + c$AI	1705	-149 *	35.6 ***	-0.14 ***	980 ***	-	0.344
	$I + aT_{sf} + bT_{sf}^2 + c$AI $+ dQ_{lf}$	119	699 ^{ns}	29.4 ***	-0.11 ***	1117 ***	-1303 **	0.438
Representative forests	$I + aT_{sf} + bT_{sf}^2$	676	809 ***	51.9 ***	-0.20 ***	-	-	0.326
	$I + aT_{sf} + bT_{sf}^2 + c$AI	675	69 ^{ns}	47.7 ***	-0.19 ***	863 ***	-	0.356
	$I + aT_{sf} + bT_{sf}^2 + c$AI $+ dQ_{lf}$	85	862 ^{ns}	35.7 ***	-0.13 ***	748 ^{ns}	-1107 ^{ns}	0.454
Grassy forest	$I + aT_{sf} + bT_{sf}^2$	191	342 ***	77.9 ***	-0.97 ***	-	-	0.545
Grassy woodland	$I + aT_{sf} + bT_{sf}^2$	103	253 ***	138 ***	4.00 *	-	-	0.581
Ash eucalypts	$I + aT_{sf} + bT_{sf}^2$	186	966 ***	22.0 ***	-0.07 ***	-	-	0.175
E. regnans	$I + aT_{sf} + bT_{sf}^2$	32	1681 ***	5.60 ^{ns}	-0.01 ^{ns}	-	-	0.029
E. pilularis	$I + aT_{sf} + bT_{sf}^2$	80	1031 ***	68.8 ***	-1.51 ***	-	-	0.434
E. diversicolor	$I + aT_{sf} + bT_{sf}^2$	148	956 ***	150 ***	-1.50 ***	-	-	0.737
E. marginata	$I + aT_{sf} + bT_{sf}^2$	353	600 ***	49.0 ***	-0.30 **	-	-	0.343
E. miniata	$I + aT_{sf} + bT_{sf}^2$	58	186 ***	109 ^{ns}	-11.46 ^{ns}	-	-	0.201
				All litter, Tsf < 40 years				
All eucalypt forests[1]	$I + aT_{sf} + bT_{sf}^2$	1596	514 ***	86.6 ***	-1.50 ***	-	-	0.273
	$I + aT_{sf} + bT_{sf}^2 + c$AI	1596	-258 ***	90.0 ***	-1.74 ***	871 ***	-	0.344
	$I + aT_{sf} + bT_{sf}^2 + c$AI $+ dQ_{lf}$	108	766 ^{ns}	129 ***	-3.02 ***	1032 ***	-1806 ***	0.593
Representative forests	$I + aT_{sf} + bT_{sf}^2$	649	589 ***	106 ***	-1.60 ***	-	-	0.359
	$I + aT_{sf} + bT_{sf}^2 + c$AI	648	434 **	106 ***	-1.75 **	1169 ***	-	0.416
	$I + aT_{sf} + bT_{sf}^2 + c$AI $+ dQ_{lf}$	78	435 ^{ns}	163 ***	-4.09 ***	934 *	-1341 *	0.650
Grassy forest	$I + aT_{sf} + bT_{sf}^2$	185	364 ***	66.6 ***	-0.45 ^{ns}	-	-	0.519
Grassy woodland	$I + aT_{sf} + bT_{sf}^2$	103	253 ***	138 ***	4.00 *	-	-	0.581
Ash eucalypts	$I + aT_{sf} + bT_{sf}^2$	170	754 ***	42.9 *	0.02 ^{ns}	-	-	0.272
E. regnans	$I + aT_{sf} + bT_{sf}^2$	21	2145 *	-100 ^{ns}	3.37 ^{ns}	-	-	0.311
E. pilularis	$I + aT_{sf} + bT_{sf}^2$	78	1036 ***	67.2 ***	-1.42 **	-	-	0.430
E. diversicolor	$I + aT_{sf} + bT_{sf}^2$	148	956 ***	150 ***	-1.50 ***	-	-	0.737

E. marginata	$I + aT_{sf} + bT_{sf}^2$	344	582 ***	59.6 ***	-0.92 **	-	-	0.221
E. miniata	$I + aT_{sf} + bT_{sf}^2$	58	186 ***	109 ^{ns}	-11.46 ^{ns}	-	-	0.201
Leaf litter only, all Tsf								
All eucalypt forests[1]	$I + aT_{sf} + bT_{sf}^2$	621	404 ***	17.5 ***	-0.08 ***	-	-	0.158
	$I + aT_{sf} + bT_{sf}^2 + cAI$	621	-40 ^{ns}	16.3 ***	-0.08 ***	512 ***	-	0.213
	$I + aT_{sf} + bT_{sf}^2 + cAI + dQ_{lf}$	81	272 ^{ns}	2.2^{ns}	-0.02 ^{ns}	327 ^{ns}	-175 ^{ns}	0.049
Representative forests	$I + aT_{sf} + bT_{sf}^2$	331	405 ***	26.8 ***	-0.12 ***	-	-	0.235
	$I + aT_{sf} + bT_{sf}^2 + cAI$	331	242 ^{ns}	25.6 ***	-0.12 ***	195 ^{ns}	-	0.239
	$I + aT_{sf} + bT_{sf}^2 + cAI + dQ_{lf}$	63	1031 ^{ns}	10.7 ^{ns}	-0.05 ^{ns}	-484 ^{ns}	-234 ^{ns}	0.072
Grassy forest	$I + aT_{sf} + bT_{sf}^2$	69	77 *	67.4 ***	-0.81 ***	-		0.718
Grassy woodland	$I + aT_{sf} + bT_{sf}^2$	76	107 **	89.8 ***	-4.13 ***	-		0.395
Ash eucalypts	$I + aT_{sf} + bT_{sf}^2$	89	469 ***	10.1 **	-0.05 **	-	-	0.104
E. regnans	$I + aT_{sf} + bT_{sf}^2$	29	886 ***	1.9 ^{ns}	-0.02 ^{ns}	-	-	0.061
E. pilularis	$I + aT_{sf} + bT_{sf}^2$	50	292 ***	13.2*	-0.25 ^{ns}	-	-	0.192
E. diversicolor	$I + aT_{sf} + bT_{sf}^2$	42	711 ***	75.6 ***	0.27 ^{ns}	-	-	0.717
E. marginata	$I + aT_{sf} + bT_{sf}^2$	162	216 ***	48.3 ***	-0.52 ***	-	-	0.493
E. miniata	$I + aT_{sf} + bT_{sf}^2$	48	138 ***	12.5 ^{ns}	3.68 ^{ns}	-		0.322
Leaf litter only, Tsf < 40 years								
All eucalypt forests[1]	$I + aT_{sf} + bT_{sf}^2$	561	179 ***	68.8 ***	-1.45 ***	-	-	0.290
	$I + aT_{sf} + bT_{sf}^2 + cAI$	561	-297 ^{ns}	70.1 ***	-1.55 ***	538 ***	-	0.342
	$I + aT_{sf} + bT_{sf}^2 + cAI + dQ_{lf}$	72	535 ^{ns}	93.0 ***	-2.50 ***	-5.85 ^{ns}	-571 ^{ns}	0.430
Representative forests	$I + aT_{sf} + bT_{sf}^2$	315	156 **	86.6 **	-1.84 ***	-	-	0.363
	$I + aT_{sf} + bT_{sf}^2 + cAI$	315	-386 **	92.7 ***	-2.15 ***	604 ***	-	0.404
	$I + aT_{sf} + bT_{sf}^2 + cAI + dQ_{lf}$	57	1704 **	135 ***	-3.67 ***	-1194 **	-781 ^{ns}	0.571
Grassy forest	$I + aT_{sf} + bT_{sf}^2$	67	55 ^{ns}	80.5 ***	-1.43 ***	-	-	0.647
Grassy woodland	$I + aT_{sf} + bT_{sf}^2$	76	107 **	89.8 ***	-4.13 ***	-	-	0.395
Ash eucalypts	$I + aT_{sf} + bT_{sf}^2$	74	336 **	34.2 ^{ns}	-0.63 ^{ns}	-	-	0.111
E. regnans	$I + aT_{sf} + bT_{sf}^2$	18	343 ^{ns}	78.8 ^{ns}	-1.88 ^{ns}	-	-	0.070
E. pilularis	$I + aT_{sf} + bT_{sf}^2$	50	292 ***	13.2 *	-0.25 ^{ns}	-	-	0.192
E. diversicolor	$I + aT_{sf} + bT_{sf}^2$	41	853 ***	110 ^{ns}	3.08 **	-	-	0.721
E. marginata	$I + aT_{sf} + bT_{sf}^2$	158	151 **	62.5 ***	-1.03 ***	-	-	0.484
E. miniata	$I + aT_{sf} + bT_{sf}^2$	48	138 ***	12.5 ^{ns}	3.68 ^{ns}	-	-	0.322

[1]Includes forests and woodlands dominated by species from genera *Eucalyptus, Corymbia, Angophora*

Supplementary Table 2. Coefficients, significance and coefficient of determination (R^2) for best-fit models using orthogonal polynomials (all models P<0.001, F-statistic) of litter accumulation (X_{Tsf} = litter mass g m^{-2}) as a function of time since fire (T_{sf}), Aridity Index (AI) and quality of litterfall (Q_{lf}). For all models I = intercept; T_{sf} = time since last fire; AI = Aridity Index. *a* and *b* are coefficients of second-order orthogonal polynoms of T_{sf}, *c* and *d* are coefficients of normal linear regression, *n* = number of observations. Also shown are models for two vegetation formations (see text). For coefficients: *** = $P < 0.001$, ** = $P < 0.01$, * = $P < 0.05$, ^{ns} = not significant (P>0.05), All coefficient P values are based on t-statistics and two-sided test.

**Community**	**Model for X_{Tsf}**	***n***	**I**	***a***	***b***	***c***	***d***	**R^2**
				Total litter				
**All eucalypt forests**	I + *poly*(T_{sf},2)	1715	1110 ***	18034 ***	-10064 ***	-	-	0.266
	I + *poly*(T_{sf},2) + *c*AI	1705	225 ***	15559 ***	-10101 ***	978 ***	-	0.344
	I + *poly*(T_{sf},2) + *c*AI + *d*Q_{lf}	119	1011 *	13617 ***	-7607 ***	1117 ***	-1303 ***	0.438
**Representative forests**	I + *poly*(T_{sf},2)	676	1280 ***	11037 ***	-11685 ***	-	-	0.326
	I + *poly*(T_{sf},2) + *c*AI	675	498 ***	9483 ***	-11215 ***	863 ***	-	0.356
	I + *poly*(T_{sf},2) + *c*AI + *d*Q_{lf}	85	1190 *	8296 ***	-7535 ***	748 ^{ns}	-1107 ^{ns}	0.454
**Grassy forest**	I + *poly*(T_{sf},2)	191	697 ***	5410 ***	-1843 ***	-	-	0.545
**Grassy woodland**	I + *poly*(T_{sf},2)	103	729 ***	4129 ***	-2125 ***	-	-	0.571
**Ash eucalypts**	I + *poly*(T_{sf},2)	186	1292 ***	3456 ***	-3204 ***	-	-	0.175
E. regnans	I + *poly*(T_{sf},2)	32	1869 ***	1002 ^{ns}	-356 ^{ns}	-	-	0.029
E. pilularis	I + *poly*(T_{sf},2)	80	1444 ***	2092 ***	-2139 ***	-	-	0.434
E. diversicolor	I + *poly*(T_{sf},2)	148	2181 ***	11957 ***	-4431 ***	-	-	0.737
E. marginata	I + *poly*(T_{sf},2)	353	928 ***	7160 ***	-1627 **	-	-	0.343
E. miniata	I + *poly*(T_{sf},2)	58	283 ***	634 **	-247 ^{ns}	-	-	0.201
				Leaf litter				
**All eucalypt forests**	I + *poly*(T_{sf},2)	621	611 ***	4010 ***	-3498 ***	-	-	0.158
	I + *poly*(T_{sf},2) + *c*AI	621	149 *	3365 ***	-2574 ***	512 ***	-	0.213
	I + *poly*(T_{sf},2) + *c*AI + *d*Q_{lf}	81	294 ^{ns}	82 ^{ns}	-802 ^{ns}	327 ^{ns}	-175 ^{ns}	0.049
**Representative forests**	I + *poly*(T_{sf},2)	331	683 ***	3361 ***	-4314 ***	-	-	0.235
	I + *poly*(T_{sf},2) + *c*AI	331	506 ***	3066 ***	-4211 ***	195 ^{ns}	-	0.239
	I + *poly*(T_{sf},2) + *c*AI + *d*Q_{lf}	63	1142 ^{ns}	1406 ^{ns}	-1674 ^{ns}	-484 ^{ns}	-234 ^{ns}	0.072
**Grassy forest**	I + *poly*(T_{sf},2)	69	345 ***	26979 ***	-1013 ***	-	-	0.718
**Grassy woodland**	I + *poly*(T_{sf},2)	76	302 ***	1047 ***	-1180 ***	-	-	0.395
**Ash eucalypts**	I + *poly*(T_{sf},2)	89	602 ***	337 ^{ns}	-2116 **	-	-	0.104
E. regnans	I + *poly*(T_{sf},2)	29	885 ***	-1049 ^{ns}	-622 ^{ns}	-	-	0.061
E. pilularis	I + *poly*(T_{sf},2)	50	384 ***	553 ^{ns}	-359 ^{ns}	-	-	0.192

E. diversicolor	I + *poly(*T_{sf},2)	42	1436 ***	9254 ***	-813 ^{ns}	-	-	0.717
E. marginata	I + *poly(*T_{sf},2)	162	502 ***	4665 ***	-2860 ***	-	-	0.493
E. miniata	I + *poly(*T_{sf},2)	48	179 ***	584 ***	79 ^{ns}	-	-	0.321

Supplementary Table 3

Coefficients, significance and coefficient of determination (R^2) for best-fit models (all models significant at P<0.001, F-statistic) of litter accumulation (X_{Tsf}, g m^{-2}) as a function of time since last fire (T_{sf}), aridity index (AI) and quality of litter (Q_l). All models are of the form $X_{Tsf} = I + aT_{sf} + bT_{sf}^2 + cAI + dQ_l$ where I = intercept, *a*-*d* are coefficients, *n* = number of replicates. Also shown are models for data aggregated by forest structure (Grassy Woodland and Grassy Forest, see Methods). For coefficients: *** = $P < 0.001$, ** = $P < 0.01$, * = $P < 0.05$, ^{ns} = not significant (P>0.05), All coefficient P values are based on t-statistics and two-sided test.

**Community**	***n***	**I**	***a***	***b***	***c***	***d***	R^2
All Eucalypt forests	618	386 ***	26.5 ***	-0.10 ***	578 ***	-173 ***	0.269
Representative forests	330	107 ^{ns}	36.0 ***	-0.14 ***	819 ***	-55 ^{ns}	0.347
All Eucalypt forests, T_{sf} < 40 years	559	-290 *	104 ***	-2.15 ***	906 ***	-112 ^{ns}	0.439
Representative forests, T_{sf} < 40 years	314	-837 ***	132 ***	-2.99 ***	1439 ***	-56 ^{ns}	0.518

Supplementary Table 4
Examples of land management and fire agency publications supporting and relying on litter (fuel) accumulation following single exponential models with a limit. The list is not exhaustive.

Publication
Reports (e.g. government agencies)
Gould J, Cruz M (2012) Australian fuel classification: Stage II. Ecosystem Sciences and Climate Adaption Flagship, CSIRO, Canberra Australia.
Watson P. (2012). Fuel load dynamics in NSW vegetation, Part 1: forests and grassy woodlands. NSW RFS report. 147.
Newnham, Glenn; Opie, Kimberley; Leonard, Justin (2017). A methodology for State-wide mapping annual fuel load and bushfire hazard in Queensland: CSIRO: EP175130
Australian Government, Department of The Environment and Energy (2019). Understanding Savanna Fire Management Methods in the Emissions Reduction Fund.
NSW Rural Fire Service (2019). Comprehensive vegetation fuel loads. NSW Government.
Bushfire and Natural Hazards CRC (2020). The prescribed burning atlas: A new system to plan effective prescribed burns. Bushfire and Natural Hazards CRC, Melbourne.
Marshall E, Kultaev D, McColl-Gausden S, Filkov A & Penman T (2021). Risk modelling for Kangaroo Island - Black Summer fires South Australia. Bushfire and Natural Hazards CRC, Melbourne.
Journal articles
Tolhurst K, Shields B, Chong D. 2008. Phoenix: development and application of a bushfire risk management tool. *Australian Journal of Emergency Management* **23**: 47–54.
Penman TD, York A. 2010. Climate and recent fire history affect fuel loads in Eucalyptus forests: Implications for fire management in a changing climate. *Forest Ecology and Management* **260**: 1791–1797.
Penman TD, Collins L, Price OF, Bradstock RA, Metcalf S, Chong DMO. 2013. Examining the relative effects of fire weather, suppression and fuel treatment on fire behaviour–a simulation study. *Journal of Environmental Management* **131**: 325-333.
Thomas PN, Watson PJ, Bradstock RA, Penman TD, Price OF. 2014. Modelling surface fine fuel dynamics across climate gradients in eucalypt forests of south-eastern Australia. *Ecography* **37**: 827 – 837
Price OF, Penman TD, Bradstock RA, Boer MM, Clarke H. 2015. Biogeographical variation in the potential effectiveness of prescribed fire in south-eastern Australia. *Journal of Biogeography* **42**: 2234-2245.
Price OF, Penman T, Bradstock RA, Borah R. 2016. The drivers of wildfire enlargement do not exhibit scale thresholds in southeastern Australian forests. *Journal of Environmental Management* **181:** 208-217.
Clarke H, Pitman AJ, Kala J, Carouge C, Haverd V, Evans JP. 2016. An investigation of future fuel load and fire weather in Australia. *Climatic Change* **139**: 591-605.
Harris RMB, Remenyi T, Fox-Hughes P, Love P, BindoffNL. 2018. Exploring the future of fuel loads in Tasmania, Australia: Shifts in vegetation in response to changing fire weather, productivity, and fire frequency. *Forests* **9**, 210.

Nyman P, Baillie CC, Duff TJ, Sheridan GJ. 2018. Eco-hydrological controls on microclimate and surface fuel evaporation in complex terrain. *Agricultural and Forest Meteorology* **252**: 49-61.
Volkova L, Roxburgh SH, Surawski NC, Meyer CP, Weston CJ. 2019. Improving reporting of national greenhouse gas emissions from forest fires for emission reduction benefits: An example from Australia. *Environmental Science & Policy* **94**: 49-62.
Collins L, Bennett AF, Leonard SWJ, Penman TD. 2019. Wildfire refugia in forests: Severe fire weather and drought mute the influence of topography and fuel age. *Global Change Biology* **25**: 3829-3843.
Price OF, Bedward M. 2020. Using a statistical model of past wildfire spread to quantify and map the likelihood of fire reaching assets and prioritise fuel treatments. *International Journal of Wildland Fire* **29**: 401-413.
Penman TD, Clarke H, Cirulis B, Boer MM, Price OF, Bradstock RA. 2020. Cost-effective prescribed burning solutions vary between landscapes in Eastern Australia. *Frontiers in Forests and Global Change* **3:** 79.
Penman TD, Cirulis BA. 2020. Cost effectiveness of fire management strategies in southern Australia. *International Journal of Wildland Fire* **29**: 427-439.
McColl-Gausden SC, Bennett LT, Duff TJ, Cawson JG, Penman TD. 2020. Climatic and edaphic gradients predict variation in wildland fuel hazard in south-eastern Australia. *Ecography* **43**: 443-455.
Clarke H, Penman T, Boer M, Cary GJ, Fontaine JB, Price O, Bradstock R. 2020. The proximal drivers of large fires: A pyrogeographic study. *Frontiers in Earth Science* **8:** 90.
Cary GJ, Blanchard W, Foster CN, Lindenmayer DB. 2021. Effects of altered fire intervals on critical timber production and conservation values. *International Journal of Wildland Fire* **30**: 322-328
Cirulis B, Clarke H, Boer MM, Penman TD, Price OF, Bradstock RA. 2021. Quantification of inter-regional differences in risk mitigation from prescribed burning across multiple management values. *International Journal of Wildland Fire* **29**: 414-426.
Clarke H, Cirulis B, Penman T, Boer MM, Bradstock RA. 2022. The 2019–2020 Australian forest fires are a harbinger of decreased prescribed burning effectiveness under rising extreme conditions. *Scientific Reports* **12:** 11871.
McColl-Gausden SC, Bennett LT, Ababi DA, Clarke HG, Penman TD. 2022. Future fire regimes increase risks to obligate-seeder forests. *Diversity and Distributions* **28:** 542-548

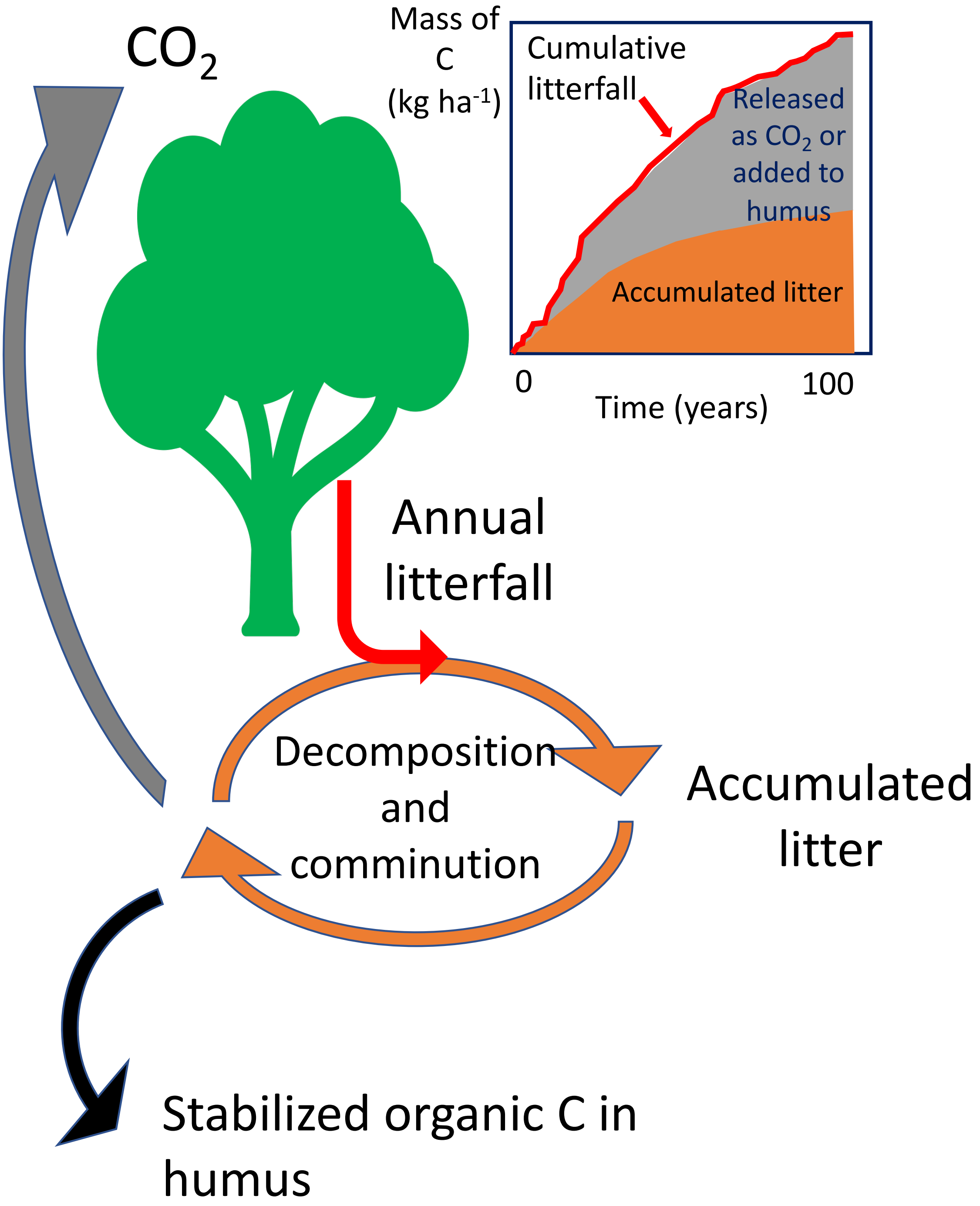

Supplementary Fig 1.
Conceptual pools and transfers of carbon involved in decomposition. Transfers of soluble C from litter to soil (e.g. dissolved organic carbon) are not represented.

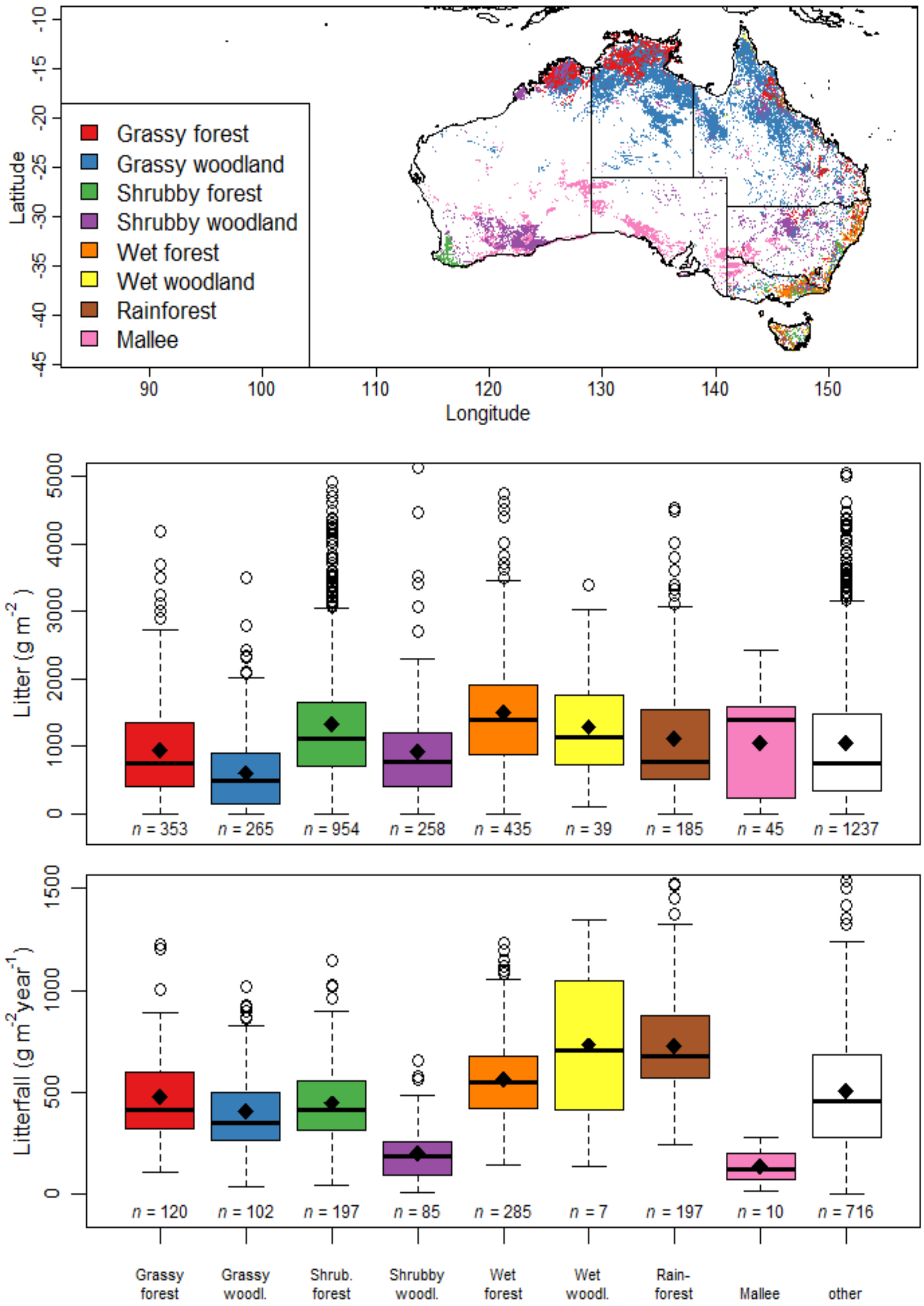

Supplementary Fig 2

Grouping of forests and woodlands into formations[54]. For each formation, summary statistical data for litter are shown in the middle panel and for litterfall in the bottom panel. Boxes represent the median, and the 25th and 75th percentiles. Whiskers extend to 1.5 times the inter-quartile range. Outliers are denoted by circles. Diamonds represent arithmetic means. The numbers of observations for each vegetation formation are shown. Base map after Neumann *et al*[69] and was prepared using R[73] software.

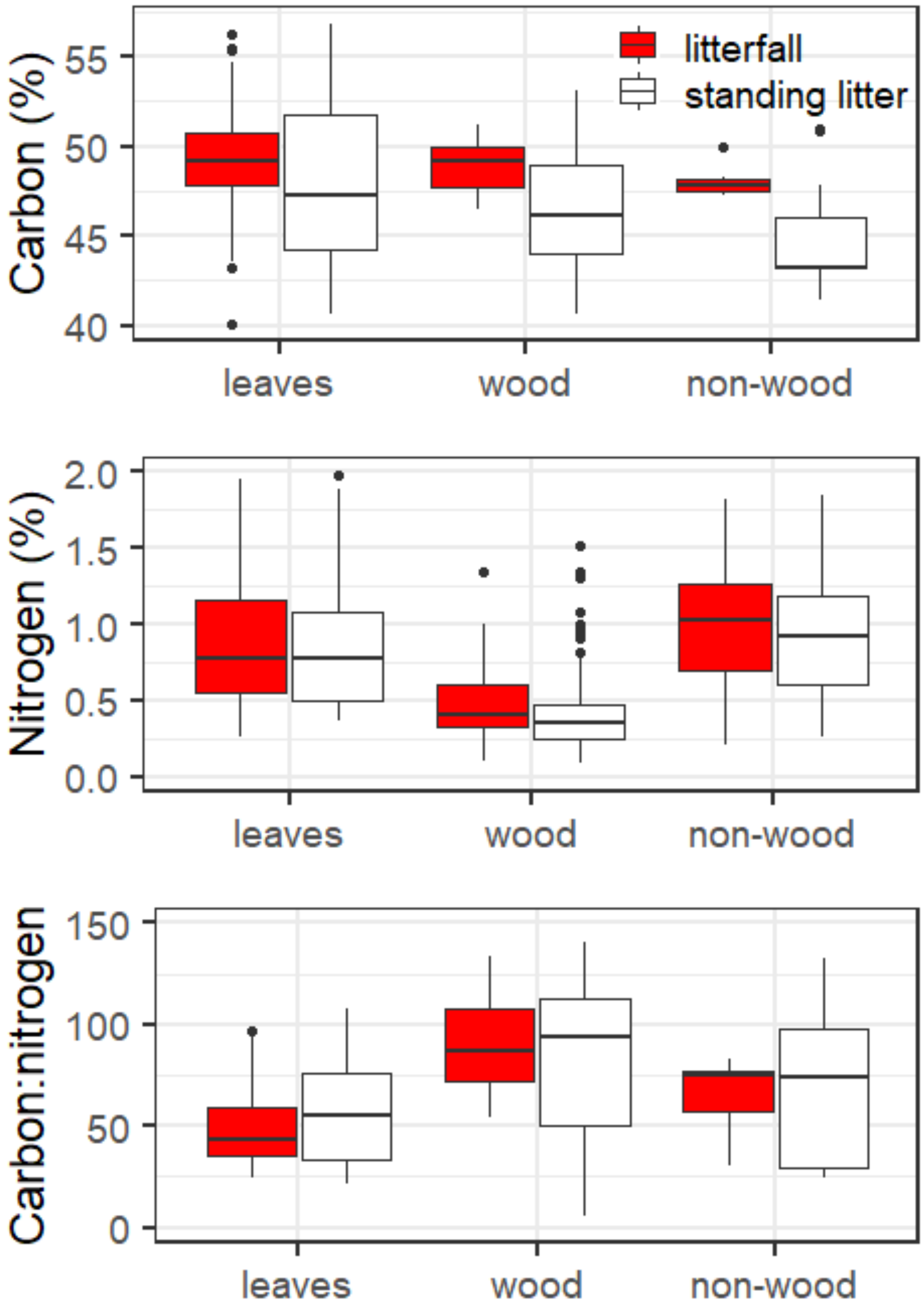

Supplementary Fig 3.
Concentrations of carbon and nitrogen in litter and litterfall components, and C:N ratios, for eucalypt forests and woodlands. Boxes represent the median and 25th and 75th percentiles, while whiskers extend to 1.5 x interquartile range. Values outside this range are indicated by dots. Number of observations (n) are as follows: litterfall carbon, leaves = 145, wood = 18, non-wood = 7; litter carbon, leaves = 107, wood = 99, non-wood = 54; litterfall nitrogen, leaves = 358, wood = 145, non-wood = 156; litter nitrogen, leaves = 146, wood = 128, non-wood = 72; litterfall C:N, leaves = 145, wood = 18, non-wood =7; litter C:N, leaves = 49, wood = 43, non-wood = 12

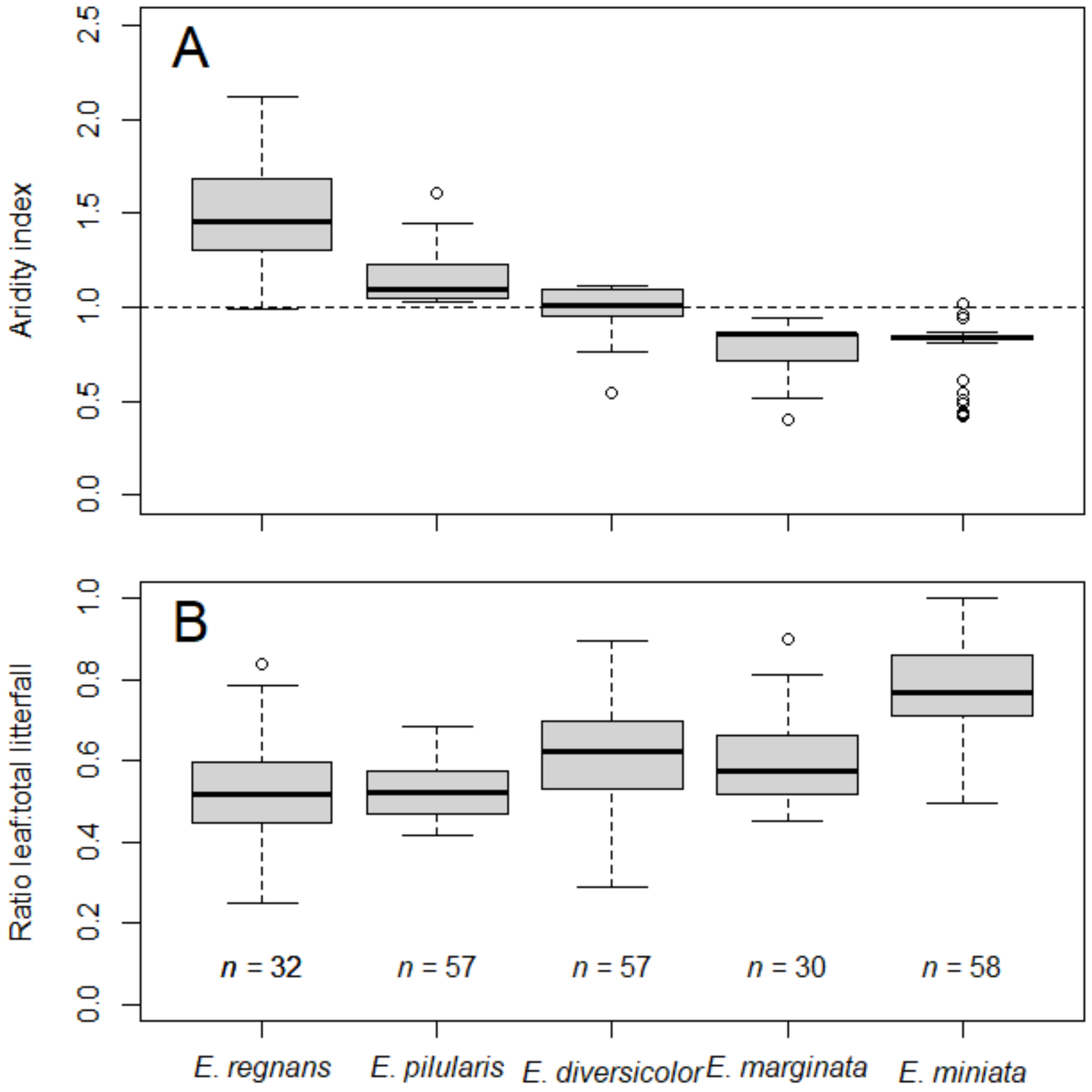

Supplementary Fig 4.
Descriptive statistics for indices of climate (panel A) and quality (panel B) for five selected forest types. In A, we indicate where Aridity Index (AI) = 1. AI> 1 indicates water surplus, AI <1 indicates a water deficit. Boxes represent the median and 25th and 75th percentiles, while whiskers extend to 1.5 x interquartile range. Values outside this range are indicated by circles.

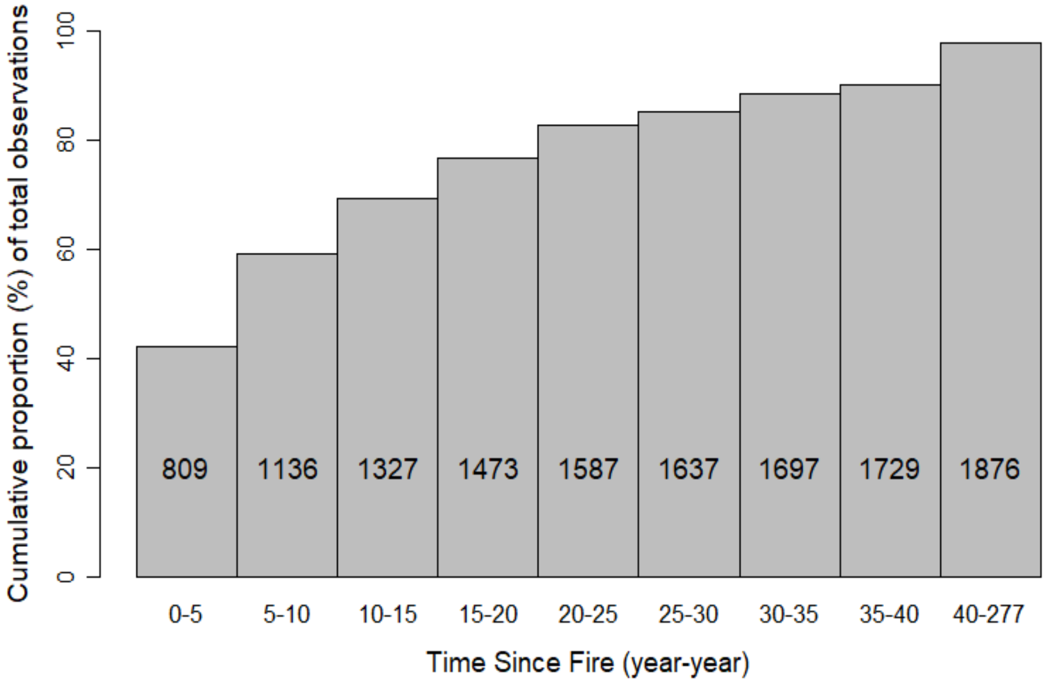

Supplementary Fig 5.
Litter data records. Data were classified according to time since fire (T_{sf}, years). 1919 studies did not record TSF. Maximum recorded T_{sf} was 277 years.

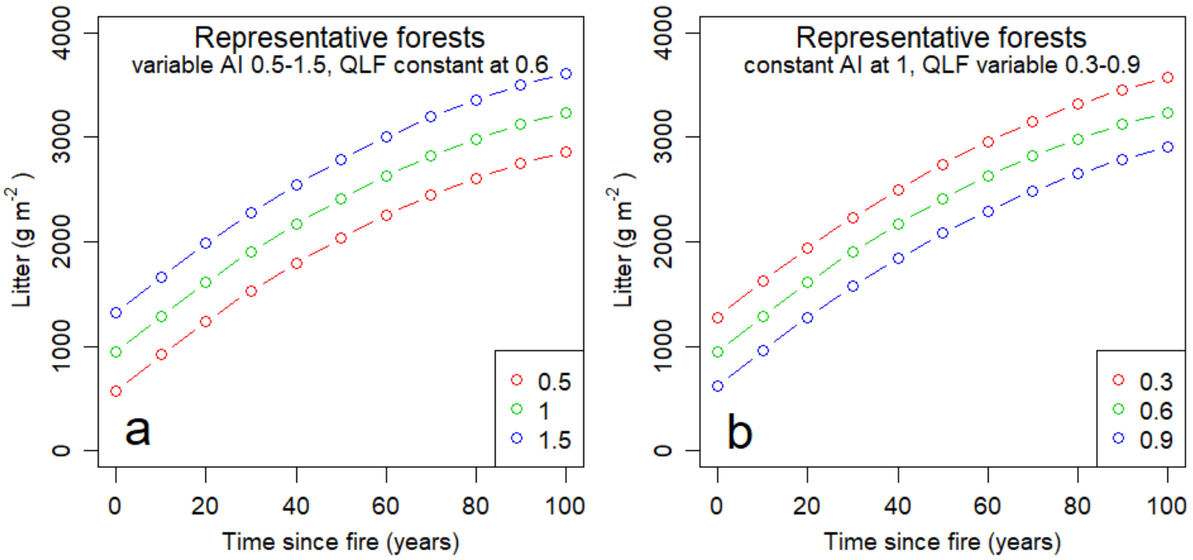

Supplementary Fig 6.
Model predictions of litter mass with increasing time (up to 100 years) for Representative forests. (A) Litter mass with fixed litterfall quality (Q_{lf}) and a range of values for aridity index (AI), (B) Litter mass with fixed AI and a range of values of Q_{lf}.
