# Supplementary material for: Litter accumulation and fire risks show direct and indirect climate-dependence at continental scale
Source: Nat Commun. 2023 Mar 18;14:1515. doi: 10.1038/s41467-023-37166-9 (PMC10024763; doi:10.1038/s41467-023-37166-9)
Supplement: Supplementary file 2 — Reporting Summary [file 41467_2023_37166_MOESM2_ESM.pdf]

nature portfolio

Corresponding author(s): MARK ADAMS

Last updated by author(s): Feb 16, 2023

Reporting Summary

Nature Portfolio wishes to improve the reproducibility of the work that we publish. This form provides structure for consistency and transparency in reporting. For further information on Nature Portfolio policies, see our Editorial Policies and the Editorial Policy Checklist.

Statistics

For all statistical analyses, confirm that the following items are present in the figure legend, table legend, main text, or Methods section.

n/a	Confirmed	
☐	☒	The exact sample size (*n*) for each experimental group/condition, given as a discrete number and unit of measurement
☐	☒	A statement on whether measurements were taken from distinct samples or whether the same sample was measured repeatedly
☐	☒	The statistical test(s) used AND whether they are one- or two-sided<br>*Only common tests should be described solely by name; describe more complex techniques in the Methods section.*
☐	☒	A description of all covariates tested
☐	☒	A description of any assumptions or corrections, such as tests of normality and adjustment for multiple comparisons
☐	☒	A full description of the statistical parameters including central tendency (e.g. means) or other basic estimates (e.g. regression coefficient) AND variation (e.g. standard deviation) or associated estimates of uncertainty (e.g. confidence intervals)
☐	☒	For null hypothesis testing, the test statistic (e.g. *F*, *t*, *r*) with confidence intervals, effect sizes, degrees of freedom and *P* value noted<br>*Give P values as exact values whenever suitable.*
☒	☐	For Bayesian analysis, information on the choice of priors and Markov chain Monte Carlo settings
☒	☐	For hierarchical and complex designs, identification of the appropriate level for tests and full reporting of outcomes
☒	☐	Estimates of effect sizes (e.g. Cohen's *d*, Pearson's *r*), indicating how they were calculated

Our web collection on statistics for biologists contains articles on many of the points above.

Software and code

Policy information about availability of computer code

Data collection	No software was used in data collection
Data analysis	Latest version R statistical software (2021) was used, and this is recorded in the reference list

For manuscripts utilizing custom algorithms or software that are central to the research but not yet described in published literature, software must be made available to editors and reviewers. We strongly encourage code deposition in a community repository (e.g. GitHub). See the Nature Portfolio guidelines for submitting code & software for further information.

Data

Policy information about availability of data

All manuscripts must include a data availability statement. This statement should provide the following information, where applicable:

- Accession codes, unique identifiers, or web links for publicly available datasets
- A description of any restrictions on data availability
- For clinical datasets or third party data, please ensure that the statement adheres to our policy

The data used here are available from Figshare:
https://figshare.com/articles/dataset/Litterfall_and_standing_litter_of_Australian_woody_ecosystems/13160351

Human research participants

Policy information about studies involving human research participants and Sex and Gender in Research.

Reporting on sex and gender	n/a
Population characteristics	n/a
Recruitment	n/a
Ethics oversight	n/a

Note that full information on the approval of the study protocol must also be provided in the manuscript.

Field-specific reporting

Please select the one below that is the best fit for your research. If you are not sure, read the appropriate sections before making your selection.

☐ Life sciences ☐ Behavioural & social sciences ☒ Ecological, evolutionary & environmental sciences

For a reference copy of the document with all sections, see nature.com/documents/nr-reporting-summary-flat.pdf

Ecological, evolutionary & environmental sciences study design

All studies must disclose on these points even when the disclosure is negative.

Study description	We collated published data on litter and litterfall. We used the data to test hypotheses that litter mass could be well described using three variables - time, litter quality and climate.
Research sample	The full data set contains more than 1812 individual datapoints for litterfall and 3887 datapoints for standing litter for the Australian continent. Data were sourced from the published literature. All available data for the Australian continent were included.
Sampling strategy	We used all data that met our criteria. Data were included if the vegetation type was eucalypt forest or woodland. We checked recorded locations against published literature.
Data collection	We extracted data from the literature. Original data were collected by a large number of authors, each fully identified in the data set. Litter samples were typically collected by hand using a frame of known area. Litterfall was typically sampled using traps of known area (e.g. mesh-bottomed frames), collected at known intervals, with sample traps moved to new locations at each sampling time.
Timing and spatial scale	The data set includes all published data 1960-2020. The spatial scale was the Australian continent.
Data exclusions	We excluded data for >40 years since previous fire owing to unreliability and likelihood of bias. This decision was made prior to analysis.
Reproducibility	We tested our models in a range of ways. We tested multiple functions (4) for goodness-of-fit. We tested use of polynoms in place of polynomial function (quadratic). We also tested alternatives to our index of quality. We report in full the outcome of these tests within the Ms.
Randomization	We used previously reported data. We did not need to randomize data. We used species identity of dominant tree species to group data. In other cases data were grouped according to vegetation formation or genetic similarity. The grouping is fully described in the methods.
Blinding	Blinding was not required as the data was used as published.

Did the study involve field work? ☐ Yes ☒ No

Reporting for specific materials, systems and methods

We require information from authors about some types of materials, experimental systems and methods used in many studies. Here, indicate whether each material, system or method listed is relevant to your study. If you are not sure if a list item applies to your research, read the appropriate section before selecting a response.

Materials & experimental systems

n/a	Involved in the study
☒	☐ Antibodies
☒	☐ Eukaryotic cell lines
☒	☐ Palaeontology and archaeology
☒	☐ Animals and other organisms
☒	☐ Clinical data
☒	☐ Dual use research of concern

Methods

n/a	Involved in the study
☒	☐ ChIP-seq
☒	☐ Flow cytometry
☒	☐ MRI-based neuroimaging
